# Artificial Intelligence for Therapeutic Peptide Discovery and Development

**DOI:** 10.3390/biom16101445

**Published:** 2026-10-03

**Authors:** Salvador Ventura

**Affiliations:** Institut de Biotecnologia i de Biomedicina, Departament de Bioquímica i Biologia Molecular, Universitat Autònoma de Barcelona, Cerdanyola del Vallès, 08193 Barcelona, Spain; salvador.ventura@uab.cat

**Keywords:** therapeutic peptides, artificial intelligence, peptide design, protein language models, generative models, molecular representation, non-canonical amino acids, active learning, benchmark integrity, translational validation

## Abstract

Therapeutic peptides combine biological specificity with chemical versatility, and artificial intelligence (AI) now contributes to their identification, design, and optimisation. Peptide discovery, however, cannot be reduced to generating amino-acid sequences: therapeutic performance also depends on chemistry, conformation, assay context, stability, delivery, safety, manufacturability, and in vivo exposure. This critical review examines AI use across the major peptide classes: receptor agonists, protein–protein interaction inhibitors, oncology and tumour-targeting peptides, antimicrobial and phenotype-driven peptides, delivery peptides, vaccine epitopes, self-assembling materials, and chemically constrained macrocycles. It compares sequence-based, chemical, graph, structure-aware, and multimodal representations across predictive, generative, and experimentally integrated workflows. Coverage spans recent general protein foundation, prediction, and design systems (ProtGPT2, ESM-2/ESM3, ProGen/ProGen2, ProteinMPNN, RFdiffusion, and AlphaFold 3) and peptide-focused methods (RFpeptides, PepFlow, and PepGLAD). Particular attention is given to benchmark integrity, the limits of structure prediction for short and non-canonical molecules, and the gap between retrospective and prospective evidence. To make claims comparable, the review adapts established reporting, validation, and readiness concepts into a peptide-specific, two-dimensional Peptide–AI Evidence Grid that separates methodological rigour (M0–M3) from translational depth (T0–T3), applies it to representative studies, and provides a checklist extending DOME, TRIPOD + AI, and FAIR guidance with the chemistry- and assay-specific items peptide work requires.

## 1. Introduction

Therapeutic peptides occupy a distinctive space between small molecules and large biologics. Like biologics, they can recognise extended and relatively flat molecular surfaces with high affinity and selectivity; like small molecules, they can often be produced by chemical synthesis and modified at defined positions. This combination makes peptides attractive for receptor activation, enzyme inhibition, and the modulation of protein–protein interactions that are difficult to address with conventional small molecules. Their intermediate size, however, does not automatically confer drug-like behaviour. Proteolysis, rapid renal clearance, aggregation, formulation instability, limited membrane permeability, insufficient tissue exposure, immunogenicity, and manufacturing complexity can all separate biochemical potency from therapeutic performance [1,2].

Peptide medicinal chemistry has developed a large repertoire of strategies to manage these liabilities. Cyclisation, hydrocarbon and other staples, disulfide engineering, backbone N-methylation, D-amino acids, terminal capping, lipidation, PEGylation, albumin-binding motifs, and payload conjugation can change the same sequence into molecules with very different conformational preferences, stability, permeability, half-life, and biodistribution [3,4]. These interventions are not peripheral annotations. They determine molecular connectivity and stereochemistry and can alter the mechanism by which a peptide reaches and engages its target. Consequently, a therapeutic peptide is often better understood as a chemically defined molecular construct than as a string drawn from the 20-letter natural amino-acid alphabet.

AI enters peptide discovery at several distinct points. Supervised models learn relationships between measured peptide properties and molecular features; protein language models transfer patterns learned from very large sequence collections; graph and chemical-language models encode atom-level chemistry and connectivity; structure-prediction and docking methods propose peptide conformations and binding modes; generative models create new sequences or chemotypes; and active-learning systems select the experiments expected to be most informative. These approaches are complementary rather than interchangeable. A sequence model can recognise motifs efficiently, for example, but it cannot infer a cyclisation bond that is absent from its input. Conversely, an atom-level graph can represent that bond but may not capture the evolutionary or contextual information learned by a protein language model.

This distinction matters because much of the apparent progress in peptide AI is still measured on retrospective benchmarks. A model may separate active from inactive entries in a curated database yet fail when applied to a new target, scaffold, laboratory, or assay. A generator may produce novel-looking sequences that satisfy its own predictors but lose activity after synthesis, prove toxic, or fall outside the chemistry on which those predictors were trained. A high-confidence predicted complex may be structurally plausible without establishing affinity, selectivity, or mechanism. The central scientific question is therefore not whether AI can assign persuasive scores, but whether it improves experimental decisions and ultimately produces better therapeutic candidates.

Two claims are developed throughout. The first is that representation and evaluation are inseparable: the molecular information a model is allowed to see determines which distinctions it can learn, and the way performance is measured determines whether an apparent gain is real. The second is that the strength of a peptide–AI study has two independent components: how rigorously the computational model was evaluated, and how far the resulting molecules were taken experimentally, and that collapsing them into a single narrative is a recurrent source of overstatement in the field. Section 9 formalises this separation as a two-dimensional evidence grid, and Section 5, Section 6, Section 7 and Section 8 apply it to predictive, generative, structural, and closed-loop work.

The review uses a class-based organisation to preserve the biological and translational distinctions that determine how peptide AI should be evaluated. It first explains why different peptide applications correspond to different computational problems. It then examines the data and molecular representations on which peptide models depend, before discussing predictive, generative, structure-aware, and closed-loop approaches. Throughout, representative studies are considered in relation to the evidence they actually provide. The final sections present the evidence grid as an operational peptide-specific synthesis of established reporting, validation, and maturity concepts, together with a reporting checklist intended to make claims more comparable and less vulnerable to overinterpretation.

This is intended to be a critical narrative, not a systematic review. The comparative tables are representative, not exhaustive: peer-reviewed studies available up to the literature cut-off (September 2026) were selected to span therapeutic classes, representation and model families, workflow types, and evidence levels, while prioritising peptide-specific methods, direct enabling technologies, sufficient reporting for M/T assessment, and prospective validation where available. General protein models are included when they materially inform peptide workflows, but protein-level validation is not credited as peptide-specific therapeutic evidence. To make the evidence base transparent, targeted searches were conducted of PubMed and publisher websites pairing therapeutic-peptide and class terms with machine-learning, deep-learning, language-model, generative, diffusion, structure-prediction, and active-learning terms, plus backward and forward citation tracing. Eligible records were peer-reviewed primary studies or enabling resources directly relevant to peptide prediction, design, synthesis, or validation and sufficiently detailed for claim-level assessment.

## 2. The Design Space

Therapeutic peptides do not define a single computational task. The object being designed may be a receptor agonist whose activity and duration must be balanced, a binder to a defined protein surface, a phenotype-inducing membrane-active peptide, a carrier for a separate cargo, an antigen intended to be processed and presented, a multicomponent conjugate, or a material whose function emerges only after self-assembly. Each case changes the relevant input variables, the meaning of the output label, and the experiment required to show success. Treating all of these problems as generic sequence-to-activity prediction obscures the biology that the model is expected to learn.

The distinction can be made concrete by asking three questions. First, what is the design object: an unmodified sequence, a chemically constrained molecule, a peptide-target complex, a peptide-cargo construct, an antigenic determinant, or an ensemble of assembling molecules? Second, what is the decision endpoint: potency at a receptor, binding to a target, selective killing, intracellular delivery, presentation and immune recognition, permeability, or a material property? Third, what observation could falsify an apparently successful prediction? Table 1 summarises these class-specific design regimes. These subsections define the class-specific context and Section 5, Section 6, Section 7 and Section 8 cross-reference them.

### 2.1. Endocrine and Metabolic Peptides

Endocrine and metabolic peptides provide one of the clearest examples of quantitative sequence-pharmacology design. Peptide hormones such as glucagon and GLP-1 activate class B G-protein-coupled receptors through distributed contacts along the ligand. A substitution at one position may alter receptor affinity, while another changes the efficiency or pathway bias of receptor activation. When a single analogue is intended to act at more than one receptor, the objective becomes a response surface rather than a single potency value: the desired candidate may need balanced activity, deliberate selectivity, or a defined ratio of activities across receptors. Binary labels such as “agonist” and “non-agonist” discard precisely the quantitative information required for this type of optimisation.

The study by Puszkarska and colleagues illustrates both the opportunity and the limits of this setting [5]. The authors trained models on experimentally measured EC50 values for 125 glucagon and GLP-1 analogues at both the glucagon receptor (GCGR) and GLP-1 receptor (GLP-1R). A multi-task convolutional model learned the two activities jointly and was then used to design 15 peptides assigned prospectively to three profiles: GCGR-selective, GLP-1R-selective, or potent at both receptors. Several designed dual agonists exceeded the potency of the training examples, whereas the GCGR-selective designs were unsuccessful, consistent with the scarcity of GCGR-selective examples in the training set [5]. The experiment is informative because success and failure can both be traced to the measured distribution: the model was effective where the sequence-function landscape was sufficiently represented and unreliable where it was not.

A complementary study used a larger medicinal-chemistry workflow to redesign secretin-derived sequences as GLP-1R agonists [6]. Systematic peptide libraries were synthesised and measured, machine-learning models linked substitutions to receptor potency and developability-related properties, and selected candidates were progressed into pharmacokinetic and in vivo studies. This staged design-synthesis-test process is closer to the way peptide leads are advanced in practice than a one-step activity classifier. It also shows why an apparently simple sequence problem is actually hierarchical: sequence changes are first translated into receptor pharmacology, then into physicochemical behaviour, and finally into exposure and efficacy.

These examples also expose an important boundary. The activity models operated within defined analogue series and chemical formats. Clinically relevant half-life extension commonly depends on modifications such as lipidation, terminal protection, or albumin binding, which can affect aggregation, absorption, protein binding, and receptor access. If these features are absent from the model input, they cannot be recovered from the sequence score. For endocrine peptides, useful AI systems should preserve receptor-specific concentration-response data, represent the complete chemical construct, and connect in vitro pharmacology to the exposure required for the intended dosing interval.

### 2.2. Target-Specific PPI and Intracellular Inhibitors

Peptide inhibitors of protein–protein interactions (PPIs) pose a different problem because the target is a defined molecular surface rather than a broad phenotype. PPIs are often mediated by extended, shallow interfaces in which a short linear motif or secondary-structure element contributes a disproportionate fraction of the binding energy. A peptide can reproduce such a motif, but its free-state conformational ensemble, orientation on the target, and competition with the native partner all influence binding. The computational task is to propose a recognition element compatible with a defined target surface and functional in the relevant biological context.

Target-conditioned language models approach this problem without requiring an experimentally determined complex. PepMLM, for example, appends a masked peptide region to the target protein sequence and fine-tunes a protein language model to reconstruct a plausible binder [7]. The model thereby learns statistical relationships between protein context and peptide sequence rather than generating peptides independently of the target. The study combined in silico evaluation with prospective binding and degradation assays, including peptides incorporated into a ubiquitin-based degrader architecture. This is an important advance, but the generated molecules were primarily canonical linear peptides, and the model’s internal likelihood or an AlphaFold-derived complex score cannot by itself establish selectivity, intracellular availability, or mechanism.

EvoBind2 uses a more explicitly structure-guided route [8]. Starting from the target sequence, it iteratively mutates a peptide and evaluates the resulting peptide-target complex with AlphaFold-based models, without requiring a predefined binding site. The method was used to design both linear and cyclic binders, and surface plasmon resonance provided prospective affinity measurements for selected candidates. The experimental results are particularly instructive because only a subset of the designed peptides bound detectably, despite favourable computational ranking. The pipeline shows that structure-prediction models can focus a difficult search, while in silico confidence functions as an enrichment signal that requires binding measurements.

Structure-conditioned generation and macrocycle design extend this logic by treating geometry as part of the design input [9,10]. Macrocyclisation can preorganize a peptide into a binding-competent state, reduce the entropic penalty of binding, and improve proteolytic stability. It can also create alternative conformers, restrict an incorrect geometry, or reduce solubility. A structure-aware generator must therefore consider not only a single bound pose but the compatibility between the free ensemble, the target-bound state, and the chosen ring topology. The success of high-affinity macrocycle design is a strong demonstration of molecular recognition, yet high affinity alone does not establish membrane permeability, systemic exposure, or activity inside cells [10].

Intracellular PPI inhibition adds a second design problem that is only partially coupled to affinity. A peptide can bind a purified target and still fail because it is degraded, trapped in endosomes, bound nonspecifically, or excluded from the relevant organelle; total cellular fluorescence is not equivalent to free cytosolic concentration. Robust programmes therefore separate four questions—does the peptide bind, is it selective, does it reach the correct compartment, and does engagement alter the expected pathway—and answer them with orthogonal biophysical assays, competition or mutational controls, direct cellular target-engagement measurements, and pathway rescue.

### 2.3. Oncology, Tumour-Targeting, and Peptide Conjugates

Oncology includes several peptide design problems that are frequently grouped under one label despite having different mechanisms. An anticancer peptide may kill cells by disrupting membranes, a tumour-homing peptide may recognise a surface receptor without being intrinsically cytotoxic, a PPI inhibitor may act on an intracellular pathway, and a peptide–drug conjugate (PDC) may use the peptide only as a targeting or retention element. A dataset that records all of these molecules as “anticancer” combines incompatible endpoints. The resulting classifier may perform well while learning little about tumour selectivity or therapeutic mechanism.

CancerPPD2 and related resources are useful for locating experimentally studied peptides, but the annotations inherit the heterogeneity of the literature: different cell lines, concentrations, exposure times, assay readouts, sequence origins, and definitions of activity [11]. Cationicity and amphipathicity, for example, can correlate with loss of cancer-cell viability because they favour membrane disruption. The same features can also produce haemolysis or toxicity in normal cells. An AI model trained without matched counter-screens may therefore rediscover general membranolytic chemistry and report it as cancer specificity. Reviews of the field have emphasised that the main limitation is no longer the absence of classifiers, but the absence of harmonised, mechanism-aware datasets and comparable prospective tests [12].

The reinforcement-learning study by Pandey and colleagues provides a useful example of phenotype-driven prioritisation at scale [13]. The method searched a focused library of approximately 36 million structurally resolved alpha-helical segments and reduced it to just over 100 peptides for experimental testing. Fifteen candidates reduced viability in a breast-cancer cell assay, and three leads showed a wider activity window relative to selected non-cancerous cell types. The campaign demonstrates substantial compression of the search space and a measurable prospective hit rate. At the same time, subsequent testing across additional breast cell lines revealed the difficulty of separating tumour selectivity from broad membrane activity. The study is therefore valuable not only as a success but also as evidence that normal-cell counter-screens and mechanistic follow-up must be designed into the objective rather than appended after ranking.

Tumour-homing peptides and PDCs require a different representation because the therapeutic object is multicomponent. A PDC contains at least a targeting peptide, a linker, a payload, and a conjugation site; each can change plasma stability, receptor binding, internalisation, intracellular release, biodistribution, and toxicity [14,15]. Two constructs with the same peptide may behave differently because one linker is cleaved prematurely, one payload prevents receptor binding, or one conjugation site changes the peptide conformation. Sequence-only models cannot distinguish these outcomes. PDCdb is valuable precisely because it organises information about peptide, linker, payload, activity, and pharmaceutical context, although source heterogeneity and related constructs must be controlled when building train-test splits [16].

A tumour-targeting programme should evaluate the final construct through receptor expression and internalisation, linker stability, payload release, tumour and normal-tissue distribution, exposure-response, and tolerability. Section 2.5 describes an ocular-delivery study that achieves this continuity outside oncology and can be read as a template for what such a chain looks like when it is complete [17].

### 2.4. Phenotype-Driven Antimicrobial and Related Peptides

Antimicrobial peptides (AMPs) are the most mature test bed for AI-assisted peptide discovery. Thousands of natural and synthetic sequences, relatively scalable activity assays, and the urgent need for new anti-infective agents have enabled supervised classifiers, quantitative MIC models, generative systems, and proteome-scale mining [18,19,20,21,22,23,24,25,26,27,28,29,30,31]. This concentration of data explains why AMPs dominate the peptide–AI literature; it does not mean that their methods or labels transfer unchanged to receptor agonists, intracellular binders, or conjugates.

The AMP endpoint is itself conditional. Minimum inhibitory concentration depends on the microbial species and strain, growth medium, inoculum, incubation time, salt content, and endpoint definition. A peptide active against one organism may be inactive against another, and an entry labelled “inactive” may simply not have been tested under a compatible condition. Species-aware models such as LLAMP make this dependence explicit by including organism context rather than learning a single universal antimicrobial label [19]. The model therefore predicts a peptide-organism relationship conditioned on assay context.

Generative AMP studies illustrate several different search strategies. deepAMP combines a pretrained peptide language model with task-specific optimisation modules and an activity predictor to optimise AMP templates [20]. In two design rounds, 29 selected candidates were synthesised and tested; most outperformed penetration in the reported bacterial assays, while selected peptides were evaluated for haemolysis, resistance development, and efficacy in a mouse wound-infection model. Across recurrent-neural-network, diffusion, and large-language-model approaches, novelty, high predictor scores, and structural plausibility are meaningful only if the selected set is synthesised and tested with host-toxicity controls [24,25,26,27]. These studies are strongest when they report the entire selected panel, not only the successful sequences, because the hit rate and failure distribution reveal how much experimental burden the model actually saves.

AMP discovery also demonstrates the value of search rather than de novo invention. Machine learning has been used to mine global and human gut microbiomes, as well as reconstructed ancient proteins, for encrypted antimicrobial sequences [28,29,30]. In these studies, the computational contribution is to rank an otherwise unmanageable biological search space. The biological claim rests on the downstream experiments: chemical synthesis, activity across relevant organisms, host–cell toxicity, mechanism, and, in selected cases, in vivo infection models. An ML-optimised peptide active against drug-resistant *Staphylococcus aureus* in mice extends the evidence beyond in vitro MIC values, but exposure, dose, formulation, and tolerability remain part of the interpretation [31].

A recurring methodological risk is that AMP models can exploit simple physicochemical shortcuts: positive sequences are often more cationic, hydrophobic, or amphipathic than negatives, close homologues appear in both partitions, and the same features drive haemolysis, so a high area under the receiver-operating characteristic curve can coexist with poor therapeutic selectivity. AMP studies provide some of the field’s most convincing prospective demonstrations, but they also show why similarity-aware splitting, matched toxicity assays, and species-specific labels are prerequisites for interpreting computational enrichment as therapeutic progress.

### 2.5. Cell-Penetrating, Oral-Delivery, and Auxiliary Functional Peptides

Cell-penetrating peptides (CPPs) differ from conventional therapeutics because transport, rather than target modulation, is their primary function. Their apparent performance depends on a system composed of the peptide, cargo, cell type, concentration, exposure time, uptake pathway, and measurement method. A sequence that delivers a small fluorophore may fail with a protein or nucleic-acid cargo; a peptide that enters one cell type may remain surface-bound or endosomally trapped in another. CPPsite 2.0 provides experimentally validated examples, but these contexts are essential parts of the label and should not be discarded during dataset construction [32].

The phrase “cell penetration” can describe several experimentally distinct events. Fluorescence associated with a cell can arise from membrane binding, internalised but endosomal material, released fluorophore, or genuinely bioavailable cytosolic cargo. Microscopy alone may not distinguish these possibilities, particularly when fixation redistributes a labelled peptide. Rigorous studies therefore combine imaging with orthogonal quantification, use surface-quenching or wash controls, compare temperatures or endocytosis inhibitors where mechanistically appropriate, and measure the activity of the delivered cargo. The relevant endpoint is the concentration of intact cargo that becomes bioavailable in its target compartment.

Machine-learning pipelines for CPPs range from retrospective classifiers to prospective multifunctional design. LightCPPgen uses an explainable predictive model and a genetic algorithm to propose sequence changes associated with penetration [33]. Such a system can generate mechanistic hypotheses and prioritise sequences, but feature attributions remain computational until controlled mutations and cargo-matched experiments test them.

The ocular-delivery study by Hsueh and colleagues shows what a more complete chain of evidence can look like [17]. Machine learning was used to engineer a peptide with three measured functions: cell entry, melanin binding, and low cytotoxicity. The selected peptide was then conjugated to brimonidine, and the complete construct was tested in rabbits, where a single injection produced prolonged intraocular-pressure lowering compared with free drug. The study’s evidence strength lies in the continuity between design objective, conjugate chemistry, tissue-retention mechanism, and in vivo pharmacology. The comparison with LightCPPgen illustrates an evidence distinction that recurs throughout this review: a computationally interpretable sequence proposal and a validated delivery system are different achievements, and both are legitimate, provided the wording matches the level reached.

Oral and transmucosal delivery impose a related but more sequential problem. A peptide must remain sufficiently stable in the formulation and luminal environment, move through mucus, cross an epithelial barrier, avoid rapid metabolic loss, and generate the required local or systemic exposure. Failure at any step can dominate the overall probability of success. Permeability measured in a single artificial or cellular system supports one component of this sequence and cannot establish oral bioavailability [1,2,34]. Models should state the barrier, assay conditions, peptide chemistry, and concentration range on which they were trained, because rankings can change across experimental systems.

For delivery peptides, the most faithful representation may include the cargo, route, formulation, and biological barrier in addition to the peptide. This requirement creates sparse, heterogeneous datasets, but simplifying the task to a context-free sequence label produces misleading certainty. AI is most useful here as a mechanism-aligned prioritisation tool: separate models may estimate proteolytic stability, mucus interaction, epithelial transport, endosomal escape, and tissue retention, while the final validation measures the complete route-specific exposure profile.

### 2.6. Vaccine, Epitope, and Immunomodulatory Peptides

Peptides intended to elicit rather than to exert a biological activity form a large and methodologically distinct class that is often omitted from peptide–AI reviews. Here the molecule is not required to bind a therapeutic target with high affinity; it must be processed, loaded onto a major histocompatibility complex (MHC) molecule, presented at the cell surface, and recognised by a T-cell receptor repertoire that differs between individuals. The design object is an antigenic determinant embedded in a host immune system, and the decision endpoint is an immune response rather than a biochemical measurement.

This class has the longest continuous history of machine learning in peptide science. MHC binding prediction moved from motif matrices to neural networks decades ago, and current pan-allele models such as NetMHCpan and NetMHCIIpan integrate binding-affinity and eluted-ligand data across thousands of alleles [35]. The Immune Epitope Database provides the curated substrate on which most of these models are trained and benchmarked [36]. The maturity of this subfield is instructive for the rest of peptide AI: allele-stratified evaluation, explicit separation of binding from elution data, and community benchmarks became standard here well before comparable practices were adopted for antimicrobial or anticancer peptide prediction.

The characteristic failure mode is nevertheless the same one described throughout this review, expressed in immunological terms. Predicted MHC binding is not presentation, presentation is not immunogenicity, and immunogenicity in one patient is not population coverage. Neoantigen prioritisation pipelines that rank thousands of candidate mutant peptides by predicted binding typically confirm only a small fraction as presented by mass spectrometry, and a smaller fraction still elicit detectable T-cell reactivity in patient-matched cells. Early clinical work on personalised neoantigen vaccination combined in silico candidate selection with mass-spectrometric confirmation of HLA presentation and demonstrated expansion of patient-specific neoantigen-reactive T cells, illustrating why predicted binding alone is insufficient [37]. Training data are additionally biased toward well-studied alleles, which means that reported accuracy can degrade sharply for under-represented populations.

Design at the construct level raises further problems that sequence scoring does not address. Multi-epitope constructs require linker choice, junctional-epitope avoidance, expression or synthesis feasibility, and adjuvant or delivery pairing; long synthetic peptides behave differently from minimal epitopes; and lipidation or conjugation to a carrier changes uptake by antigen-presenting cells. Immunomodulatory peptides that act on immune receptors directly, rather than as antigens, belong to the target-specific class of Section 2.2 and should not be pooled with epitope prediction. The evidential requirement for a vaccine claim is therefore layered: predicted binding (computational), confirmed presentation (immunopeptidomics), confirmed reactivity (patient-derived or transgenic T-cell assays), and finally in vivo immunisation with a measured, durable, and appropriately controlled response.

### 2.7. Self-Assembling Peptides and Peptide Materials

Self-assembling peptides shift the design target from the behaviour of one molecule to the collective behaviour of many molecules. A short sequence may form fibres, tapes, micelles, or hydrogels depending on concentration, pH, ionic strength, solvent, temperature, and preparation history. The relevant output can be assembly propensity, morphology, gelation, rheology, cargo release, or biological function. These are related but not interchangeable labels, and a model trained to predict whether a peptide forms a gel cannot automatically predict the mechanical or therapeutic properties of that gel.

This class is well suited to experimentally coupled learning because the combinatorial sequence space is large while each experiment can provide a clear, if condition-dependent, phenotype. Batra and colleagues showed that machine learning could identify self-assembling sequences outside the motifs favoured by human intuition [38]. Xu and colleagues used iterative exchange between experiment and modelling: more than 160 tetrapeptides were synthesised, the data were used to refine a gelation score, and the resulting model was applied to an 8000-sequence library, yielding a high reported success rate and a hydrogel tested as an immunological material in mice [39]. Generative approaches have subsequently combined sequence models, hybrid descriptors, and experimental validation to navigate broader assembly landscapes [40].

The scientific challenge is multiscale. Local side-chain interactions and backbone hydrogen bonding determine early association, but nucleation, fibre growth, network formation, and bulk mechanics occur over larger spatial and temporal scales. Atomistic or coarse-grained simulations can explain selected steps, yet their force fields, system size, and timescale define a strict domain of validity. For self-assembling peptides, the environment is part of the molecular input, and experimental morphology, kinetics, and material properties remain indispensable. The design object is an assembly process that produces a defined material state under specified conditions.

Table 2 presents representative approved peptide therapeutics in a residue-level notation that preserves stereochemistry, terminal chemistry, covalent connectivity, lipidation, and conjugated payloads.

## 3. Data Foundations and Benchmark Integrity

Peptide AI is constrained less by the availability of model architectures than by the quality and meaning of the data used to train them. Most datasets are assembled from publications, patents, databases, or project-specific screening campaigns. These sources were generated for different purposes and often use different assay formats, units, thresholds, controls, and reporting conventions. Pooling them increases sample size but can create labels that appear uniform while describing biologically different experiments. A model trained on such a collection may learn which laboratory, peptide family, or publication style produced an entry rather than the underlying property of interest.

The problem begins with molecular identity. The same displayed sequence can refer to a free acid, an amide, an acetylated peptide, a disulfide isomer, a cyclic construct, or a labelled assay reagent. Stereochemistry, crosslink position, salt form, and conjugation may be absent or stored in non-standard text fields. Exact sequence deduplication therefore does not guarantee chemical deduplication. Conversely, two records with different textual names may describe the same molecular species. Data cleaning for peptide AI must canonicalise both sequence and chemistry while retaining provenance, because conflicting measurements are often scientifically informative rather than simple errors.

Endpoint harmonisation is equally important. Potency can be expressed as EC50, IC50, Kd, Ki, MIC, percentage inhibition at one concentration, or a qualitative activity class. These values are not freely interchangeable. Censored results such as “>100 micromolar” contain interval information and should not be converted to an arbitrary point value without sensitivity analysis. Replicates, assay precision, species or cell context, and protocol variables should be retained whenever possible. For classification, the threshold that defines activity should be justified biologically and kept separate from the final test set; otherwise, threshold tuning becomes another form of overfitting.

Negative data require particular care. There is no universal inactive peptide: inactivity is conditional on target, organism, cell type, concentration, route, and assay. Unlabelled sequences are not reliable negatives, and random decoys can be so different from positives that the model solves an artificial discrimination problem. More informative negatives include experimentally inactive analogues, activity cliffs, scrambled controls, homologous targets, normal-cell counter-screens, and compounds that failed for known developability reasons.

A structural limitation of the public landscape deserves explicit mention. The largest and most internally consistent peptide datasets, matched analogue series measured in one laboratory under one protocol, with complete failure records, are held by pharmaceutical companies and are largely unpublished. Public resources are correspondingly enriched in positive results and in sequences that someone chose to publish. This selection bias sets a ceiling on what open benchmarks can demonstrate, and it will not be lifted by aggregating more literature. Precompetitive data-sharing consortia and federated or privacy-preserving learning, in which models are trained across institutional boundaries without moving the underlying data, offer the most plausible route to datasets whose negative space is realistic; comparable efforts in small-molecule discovery provide both a template and a record of the practical difficulties involved [50]. Table 3 summarises representative public resources and the specific questions for which they are useful.

### 3.1. Split Design and External Validation

A data split is not a technical afterthought; it defines the scientific claim being tested. A random split asks whether the model can interpolate among examples drawn from the same mixed collection. This may be appropriate for quality control within a mature chemical series, but it does not show that the model can extrapolate to a new scaffold, target family, or laboratory. When close analogues are distributed across training and test sets, apparent test performance can largely reflect recognition of a known series.

Sequence-cluster splits reduce this problem by keeping related peptides in the same partition. For short peptides, however, percentage identity is an imperfect measure: a difference in one or two residues can produce a large percentage change, while a short shared motif may dominate function despite low global identity. Sequence identity should therefore be supplemented by alignment length, motif overlap, physicochemical similarity, and, for modified peptides, chemical scaffold. Macrocycles and conjugates require topology- or scaffold-aware clustering because two molecules with different residue strings may share the same ring system, linker, or payload and remain near-duplicates from a medicinal-chemistry perspective.

Time splits provide a different test. Training on studies available before a specified date and evaluating on later data more closely reproduces the intended use of a model, although changes in assay practice and research priorities can complicate interpretation. Target-family or organism holdouts are useful when the claim concerns transfer to unseen biological contexts. External validation should use a genuinely independent source and must be deduplicated against all training and model-development data at the sequence, scaffold, target, and publication levels. A second database is not independent if it imports many of the same records.

No universal similarity threshold can capture every peptide class, and this review does not propose one. What can be standardised is the reporting: a study should declare a primary split, state the similarity criterion and threshold used to construct it, repeat the evaluation at two less stringent thresholds as a sensitivity analysis, and report the nearest training neighbour of every test or generated candidate. Where a conventional value is helpful as a starting point, 30% identity with sensitivity analyses at 50% and 70% is a defensible default for canonical linear peptides, but it should be presented as a declared choice rather than a standard, and for very short or chemically modified peptides the justification of the similarity measure matters more than any numerical cut-off. PepBenchmark is a useful emerging effort to standardise datasets, preprocessing, and baselines across canonical and non-canonical peptide tasks [54]. It was presented as a peer-reviewed poster at ICLR 2026; nevertheless, each endpoint has its own sources of relatedness, so participation in a shared benchmark should complement, not replace, task-specific cluster, scaffold, time, and external evaluations.

### 3.2. Leakage, Reproducibility, and Negative Data

Data leakage occurs whenever information from the nominal test set influences training, representation, model selection, or candidate choice. Direct duplicate sequences are the most visible form, but leakage can also arise through overlapping databases, analogue series, repeated measurements of the same construct, augmentation before splitting, target homologues, or manually selected examples. In small peptide datasets, even a few related compounds can substantially alter performance estimates.

Pretrained models introduce a less tractable form of overlap. Protein language models may have seen natural sequences related to a test peptide or its target during pretraining, and the exact composition of very large corpora can be difficult to reconstruct. This does not invalidate transfer learning, but it changes the interpretation: the downstream study should disclose the pretraining corpus and perform nearest-neighbour and homology analyses where possible. Claims of de novo generalisation should be conservative when training exposure cannot be excluded.

Reproducibility requires more than releasing a model checkpoint: the dataset version, chemical canonicalization, split membership, seeds, preprocessing code, and hyperparameter-selection procedure all determine whether a number can be regenerated, and the test set must remain frozen until the analysis plan is complete. These requirements are not specific to peptides, and Section 10 relates them to the general reproducibility standards already established for machine learning in the life sciences [59,60].

Prospective failures are especially valuable data. Inactive peptides, cytotoxic peptides, insoluble candidates, unexpected assay artefacts, and synthesis failures define the boundaries of the design space more directly than successful examples alone. Closed-loop studies should report every peptide selected for synthesis and every manual exclusion made after model ranking. Without this record, the contribution of the algorithm cannot be separated from unreported expert triage, and the literature accumulates an unrealistically favourable view of model calibration.

## 4. Molecular Representation: What Must a Peptide Model Encode?

A molecular representation is the form in which a model is allowed to see a peptide. It determines which distinctions the model can learn and which are impossible because the relevant information has been discarded. The standard one-letter amino-acid sequence is compact and biologically meaningful for canonical linear peptides, but it does not uniquely specify stereochemistry, terminal chemistry, branch points, crosslinks, staples, non-peptidic linkers, or payload attachment. For many therapeutic peptides, these features are part of the molecule rather than optional metadata.

There is no universally superior representation. Sequence representations are efficient and exploit patterns learned from natural proteins; chemical graphs preserve atoms, bonds, and stereochemistry; three-dimensional models describe geometry; and multimodal systems combine these views with target and assay context. The correct choice follows from the endpoint. A model that predicts receptor activity within a canonical analogue series may need little more than sequence and assay metadata. A model that compares N-methylated macrocycles or PDCs must represent the actual chemistry and connectivity.

### 4.1. Sequence and Protein-Language Representations

Conventional sequence models treat a peptide as an ordered series of residue tokens. One-hot encodings represent each amino acid as an independent category, whereas learned embeddings place residues and sequences in a continuous numerical space. Protein language models extend this idea by training on millions of natural protein sequences using tasks such as predicting masked residues, and during pretraining they learn statistical regularities associated with motifs, secondary structure, evolutionary constraints, and functional context [61,62,63,64]. The resulting embeddings can then be fine-tuned for a smaller peptide dataset. This transfer is attractive because most peptide property datasets are too small to train a large model from the beginning. PeptideBERT and PepLand illustrate efforts to adapt transformer-based representation learning more directly to peptide space [65,66]; such models recognise context-dependent sequence patterns, often outperform handcrafted descriptors on retrospective tasks, handle variable-length sequences, and support generation through masked or autoregressive decoding.

Within the general protein-model landscape, ESM-2 learns masked-sequence representations and underlies ESMFold, ESM3 jointly models sequence, structure, and function, and ProtGPT2 and ProGen/ProGen2 perform autoregressive protein-sequence modelling and generation [63,64,67,68,69]. These systems provide important transferable priors, but they were trained and primarily validated on proteins; their application to short or chemically modified therapeutic peptides therefore requires peptide-specific evaluation.

The main limitation is domain mismatch. Natural proteins are long, genetically encoded polymers shaped by evolution, whereas therapeutic peptides are often short, synthetic, heavily modified, and deliberately outside natural sequence distributions. A language model can assign an embedding to a 12-residue peptide, but the biological meaning of that embedding may be weak if the peptide lacks the evolutionary context present during pretraining. Short length also makes small compositional biases or shared motifs disproportionately influential.

Vocabulary extension can add tokens for selected non-canonical amino acids or modifications. This is practical within a bounded chemistry campaign, but a token is only a label unless the model is given information about the monomer’s structure. Chemically similar residues may be treated as unrelated categories, rare tokens have little training support, and unseen monomers remain out of vocabulary. Moreover, a token sequence does not necessarily specify which residues are connected by a staple or cyclisation bond. Sequence models are therefore powerful for motif and analogue learning, but they should not be described as chemistry-complete unless stereochemistry and topology are encoded explicitly.

### 4.2. Chemical Languages, Graphs, and Atom-Level Models

Chemical representations describe the peptide as a molecule rather than as a biological string. SMILES expresses atoms and bonds as a line notation, SELFIES provides a syntax designed to generate valid molecular strings, and molecular graphs represent atoms as nodes connected by bonds. These formats can encode D-residues, N-methylation, terminal caps, lipid chains, linkers, crosslinks, and other non-canonical features, provided that stereochemistry and attachment points are preserved. Atom-level geometric networks can additionally use three-dimensional coordinates and local chemical environments.

HELM occupies an intermediate level that is particularly relevant to peptide medicinal chemistry [70]. Instead of writing every atom explicitly, it represents a macromolecule as defined monomers connected through specified attachment points. A chemist can therefore distinguish monomer identity from polymer topology and describe complex constructs more transparently than with a plain residue alphabet. HELM-GPT demonstrates how this notation can be used as the language of a generative model for macrocyclic peptides [71].

Chemical representations solve some ambiguities but introduce others. SMILES is not unique unless canonicalised; protonation state, tautomerism, salt form, aromaticity, and stereochemical conventions must be standardised; and a graph contains no conformational ensemble unless coordinates are added. Preprocessing should preserve the distinction between the neutral molecule used in a calculation, the ionic state present in an assay, and the administered salt or formulation. Chemical graphs are also data-hungry and may lose useful biological priors. A graph neural network sees local atomic neighbourhoods but does not automatically know that a sequence motif is associated with a receptor family or protease recognition site. Hybrid systems can combine protein-language embeddings with residue- or atom-level graphs, allowing the model to use both sequence context and chemistry. PeptideCLM is one example of a peptide-aware chemical language model applied to cyclic-peptide membrane diffusion [72]. Such hybrids are promising, but ablation studies are needed to show whether the chemical modality improves genuine transfer rather than merely identifying the source dataset or scaffold family.

### 4.3. Topology and Specific Chemical Modifications

Non-canonical chemistry should be represented at the level at which it changes the molecule. For a modified residue, this includes monomer structure, stereochemistry, charge state, and connection points. Mapping a D-amino acid to the corresponding L-residue erases a geometric difference that can alter proteolysis, receptor recognition, and membrane transport. Mapping an N-methylated residue to its unmethylated parent removes a change in hydrogen-bonding capacity and backbone conformational preference. When an unseen monomer is encountered, the model should flag it as outside the represented domain rather than silently substitute a canonical analogue.

Terminal chemistry is equally consequential for short peptides because the termini contribute a large fraction of the total charge and hydrogen-bonding surface. Amidation, acetylation, alkylation, fluorescent labels, and conjugation can change activity, solubility, uptake, and assay readout. Models should distinguish the therapeutic construct from an assay probe; a fluorophore-labelled CPP, for example, may have different membrane interactions from the unlabelled peptide whose delivery is being inferred.

Crosslinks define topology. Disulfides, lactams, head-to-tail cyclisation, side-chain closures, and hydrocarbon staples can produce several constitutional or stereochemical isomers from the same residue list. The closure bond, redox state, staple type, positions, and linker geometry must therefore be explicit, because ring topology determines which conformations are accessible. A sequence-only model that knows a peptide is “cyclic” but not how it is closed cannot distinguish molecules with different ring sizes or contact patterns [3,4,58]. Lipidation, PEGylation, albumin-binding motifs, and payload conjugation create composite systems in which the modification may dominate pharmacokinetics, aggregation, membrane association, and tissue distribution, and its effect depends on the attachment site and linker. These molecules should be represented jointly rather than by predicting the unmodified peptide and adding an informal correction; the same principle applies to PDCs, where peptide, linker, and payload form one therapeutic object.

Task-specific models show what is possible when chemistry is defined carefully. CamSol-PTM extends solubility prediction to a specified set of modified amino acids by estimating their physicochemical contributions and validating selected peptides experimentally [73]. PEPstrMOD provides a route to structural modelling for natural, non-natural, and modified residues [74]. These tools increase the tractable chemical space, but neither constitutes a universal solution. Their predictions are reliable only for represented modifications, endpoints, and modelling assumptions, and larger moieties such as lipids or complex glycans may require a different representation.

### 4.4. Three-Dimensional Ensembles and Multimodal Representations

A peptide is not a single static structure. Many linear peptides are disordered in solution and adopt a defined conformation only on binding. Macrocycles may interconvert among several low-energy states, and the populations can change between water, membrane-like environments, and a protein pocket. Properties such as passive permeability can depend on transient intramolecular hydrogen bonds that shield polar groups in one environment and expose them in another. A single energy-minimised conformer can therefore be a poor summary of the molecule.

Ensemble representations describe multiple conformations together with their relative energies or probabilities. CREMP contains tens of thousands of macrocycles and millions of computed geometries, providing a resource for learning relationships between chemical structure, conformational diversity, and permeability [58]. The value of such data is not that every computed conformer is experimentally populated, but that models can be trained to reason about distributions rather than one arbitrary pose. Experimental NMR, crystallography, hydrogen-deuterium exchange, or permeability measurements remain important anchors for assessing whether the simulated ensemble is realistic.

Multimodal models can combine sequence embeddings, chemical graphs, conformational ensembles, target structures, assay metadata, and formulation variables. This is conceptually appropriate because therapeutic performance arises from interactions among these layers. It is also technically demanding: some modalities will be missing for many records, and the presence of a structure or a particular assay may reveal the dataset source. Models should be evaluated under realistic missing-data conditions and compared with simpler baselines to determine whether each modality contributes transferable information.

The practical goal is not to collect every possible representation but to match information to the decision. Sequence plus receptor identity may be sufficient for ranking close endocrine analogues; a macrocycle-permeability model may need atom-level chemistry and an ensemble descriptor; a target-specific intracellular inhibitor may require target context, binding geometry, cell-entry information, and assay conditions. Representation should therefore be justified prospectively: the authors should state which molecular distinctions matter for the intended use and show that the chosen encoding preserves them.

## 5. Predictive Modelling: Activity, Developability, and Safety

Predictive models estimate a measured property for candidates that already exist in a library or proposed design set. Classification assigns categories such as active or inactive, whereas regression estimates continuous values such as EC50, MIC, solubility, or permeability. Ranking models may be useful even when absolute prediction is poor, but they answer a narrower question: which candidate should be tested first within a defined pool? These outputs should not be conflated, and performance metrics should reflect the decision the model is intended to support.

In peptide discovery, a prediction is always conditional on chemistry, assay, and biological context. A model does not estimate an intrinsic truth about a sequence; it estimates what is likely to be observed under conditions represented in its data. The most useful predictors therefore sit inside a measured funnel. They remove obvious liabilities, prioritise informative candidates, and state when a molecule lies outside the domain where the prediction has been tested. Table 4 summarises representative predictive and property models together with the evidence level each publication reached, using the notation defined in Section 9.

### 5.1. Target Activity, Peptide-Protein Interactions, and Model Reliability

Target-activity models can be built at several levels. A sequence-only model predicts activity from the peptide, implicitly averaging over target and assay context. A peptide-target model takes both partners as input and can ask whether a given peptide interacts with a particular protein. Structure-aware models additionally consider contacts or geometry. CAMP, for example, uses peptide and protein sequence information to predict interactions and binding residues [75]. Such models are useful for screening, but their performance depends strongly on how non-interacting pairs are constructed and whether homologous targets or peptide families occur in both training and test sets.

For receptor pharmacology, the label should preserve the concentration-response experiment. EC50 reflects potency but can be affected by receptor expression, signal amplification, and assay pathway; efficacy describes the maximum response; and biased agonism requires comparison across signalling outputs. Collapsing these measurements into one binary label removes information needed to design partial agonists, balanced co-agonists, or pathway-selective ligands. The multi-receptor studies discussed above show the advantage of learning related outputs jointly when the same sequence influences several pharmacological dimensions [5,6].

For PPI inhibitors, binding and function must remain separate labels. A peptide can bind a protein without competing with the native partner, and it can alter a cellular phenotype through an unintended target. Conversely, a weak but highly localised interaction may produce function in cells. Affinity models should therefore be evaluated with negative targets and homologous interfaces, while functional models should include mechanistic controls. Predicted contact maps or complex structures can help formulate these tests but do not replace them.

The strongest target-specific evidence combines orthogonal methods that establish binding through different physical observables, mutations that test the proposed interface, and cellular assays that show whether binding occurs in the relevant compartment. This matters especially for AI-designed peptides, because a model can exploit assay-specific correlations that are invisible when only one readout is used.

Target-activity predictions should be reported with calibrated uncertainty and a declared applicability domain. For classification, reliability diagrams, Brier scores, or expected calibration error test whether probabilities correspond to observed frequencies; for regression, prediction intervals require both coverage and width. Domain definitions should reflect the intended use and may include sequence or chemical similarity, target family, residue alphabet, peptide length, topology, and assay conditions. Reporting each selected candidate’s nearest training neighbour, local performance, and any unseen monomer or topology makes extrapolation explicit [57]. Conformal prediction, ensembles, Bayesian approximations, and distance-based methods can quantify uncertainty but do not remove distribution shift. Selective prediction provides an operational response: the model abstains when uncertainty is high, and candidate selection uses uncertainty prospectively to distinguish exploitation from justified exploration.

### 5.2. Solubility, Aggregation, Stability, and Permeability

Developability properties determine whether an active peptide can be prepared, formulated, delivered, and maintained at an effective concentration. Solubility and aggregation are related but not identical: a peptide can have low equilibrium solubility without forming ordered aggregates, or it can be nominally soluble while producing time-dependent oligomers or particles. Measurements depend on pH, ionic strength, temperature, concentration, counterion, container surface, and formulation. Models should therefore predict a defined assay endpoint rather than the generic concept of “solubility”.

Sequence-based aggregation predictors such as AGGRESCAN identify local sequence segments associated with aggregation in canonical polypeptides [76,77]. They remain useful for early triage and for explaining how hydrophobic or beta-prone regions contribute to self-association. Transfer to short, cyclic, or heavily modified peptides is not automatic because termini, crosslinks, and non-canonical residues can dominate behaviour. A prediction developed for disease-related proteins should not be presented as a validated formulation model for synthetic peptides without direct testing. CamSol-PTM provides a more peptide-specific example: the method estimates the contribution of modified amino acids to intrinsic solubility using computed physicochemical descriptors, screened more than 10,000 variants in selected peptide series, and experimentally tested designed subsets [73]. It also defines its own boundary, since intrinsic solubility and amino-acid-scale modifications do not encompass formulation concentration, lipidated constructs, or exposure in plasma.

Proteolytic stability is similarly context-dependent. Cleavage-site predictors can identify sequence motifs recognised by selected proteases and support construction of task-specific models, as exemplified by ProsperousPlus [78]. Yet resistance in one enzyme assay may not predict stability in plasma, intestinal fluid, tissue homogenate, or intracellular compartments. D-residues, N-methylation, cyclisation, and terminal protection may block one cleavage pathway while changing permeability or target binding. Stability models are most informative when the protease panel and intended route are specified.

Permeability is one of the most difficult peptide properties because it depends on both chemistry and conformational dynamics. Cyclic peptides may cross membranes through environment-dependent conformations that alternately expose and shield polar groups. CycPeptMPDB and associated applicability-domain analyses illustrate the value of task-specific experimental data and the risk of evaluating related scaffold series with random splits [56,57]. A predictor trained on one barrier or assay cannot be assumed to estimate oral absorption, cell entry, or blood–brain barrier transport. Route-relevant experiments and explicit domain analysis are essential.

### 5.3. Manufacturability and Regulatory Constraints

Developability for peptides includes a manufacturing dimension that computational studies rarely model. Solid-phase synthesis yield falls with length and with aggregation-prone or sterically hindered sequences; difficult couplings, epimerization, aspartimide formation, and incomplete deprotection generate impurities that are structurally similar to the product and therefore hard to remove; macrocyclisation competes with oligomerisation and is concentration-dependent; and orthogonal protection schemes for multiply bridged peptides can make an otherwise attractive design impractical. A generator that proposes sequences without regard to these constraints will produce candidates whose experimental cost is systematically underestimated. CycloPepper is notable precisely because it treats cyclisation outcome as a learnable objective and validates predictions prospectively [79], and the same logic should be extended to coupling difficulty, purification burden, and cost of goods. Environmental considerations reinforce the point, since conventional synthesis is solvent-intensive and greener routes constrain the reagents and chemistries available [80].

Regulatory expectations bound the design space in ways that are also absent from most models. In July 2026, the U.S. Food and Drug Administration published 17 revised draft product-specific guidances for generic peptide products. The updated recommendations address the route of peptide manufacture used for an abbreviated new drug application, innate immune response testing, impurity thresholds, higher-order structure, and biological activity; the agency simultaneously withdrew its 2021 general guidance because it no longer reflected current scientific thinking [81]. These developments have direct consequences for AI-assisted design. A modification that improves predicted stability may generate a difficult-to-resolve diastereomer; a non-canonical residue may lack an established analytical method; and a design that is one deletion sequence away from a potentially immunogenic impurity can create a specific development risk. None of these considerations is captured by potency, permeability, or toxicity scores. Incorporating manufacturability, analytical control, and impurity-related constraints as explicit objectives, rather than as post hoc filters, is therefore one of the clearest opportunities for peptide AI to affect real development timelines.

### 5.4. Toxicity, Immunogenicity, and Off-Target Liabilities

“Peptide toxicity” is not one transferable endpoint. Membrane-active peptides can lyse erythrocytes or damage mammalian cells; receptor agonists can activate related receptors; PPI inhibitors can bind homologous interfaces; conjugates inherit payload and linker toxicities; and repeated systemic administration can introduce immunogenicity risks. Combining these mechanisms in one training label produces a model that is difficult to interpret and unlikely to support a specific medicinal decision.

ToxinPred2 illustrates the importance of naming the task correctly [82]. It was developed as a predictor of protein toxicity and can serve as a broad sequence-level screen, but it is not a validated substitute for peptide-specific haemolysis, cytotoxicity, immunostimulation, or off-target pharmacology. Differences in sequence length, mechanism, and label definition limit direct transfer. A generic toxicity score may remove obvious hazards, but it cannot establish a therapeutic window.

Safety evaluation should follow the class-specific mechanisms defined in Section 2.1, Section 2.2, Section 2.3, Section 2.4, Section 2.5 and Section 2.6. AMP studies should pair microbial activity with haemolysis and mammalian-cell toxicity; anticancer programmes should include relevant normal cells; CPP studies should distinguish productive uptake from membrane damage; and receptor ligands and PPI inhibitors should use biologically plausible counter-target panels.

Immunogenicity is especially difficult to predict from peptide sequence alone. The MHC-binding models discussed in Section 2.6 can identify potential T-cell epitopes, and they are the same tools used for vaccine design, but here the objective is inverted: the aim is to avoid what a vaccine seeks to elicit [35]. Immune response also depends on route, dose, aggregation, formulation, patient genetics, and tolerance to endogenous homologues, and chemical modifications may reduce proteolysis while creating new antigenic determinants or altering processing. Immunogenicity prediction should therefore be treated as risk assessment, with uncertainty and population coverage made explicit, rather than as a binary declaration of safety.

**Table 4:** Representative predictive and property models for therapeutic peptides, with evidence level as defined in Section 9.

Method	Peptide Class	Representation and Training Data	Task and Split Strategy	ncAA/Topology Handling	Evidence (M/T)	Code/Repository	Principal Limitation
***General prediction and property models***							
PeptideBERT	Multiple canonical peptide properties	Residue tokens; transformer trained on peptide property datasets	Supervised property prediction; task-specific retrospective splits	Limited; standard alphabet unless extended	M0/T0	GitHub Code/data scripts; task-fine-tuned weights not located	Useful transferable baseline, but chemical topology and assay context are not intrinsic to the representation [65]
PepLand	Canonical and non-canonical peptide space	Chemistry-aware peptide pretraining on a large corpus	General representation learning; downstream task-dependent splits	Broader ncAA coverage than protein-only alphabets	M1/T0	GitHub Code/checkpoints/evaluation data	Coverage depends on encoded monomers and downstream labels; split details must be inspected per task [66]
CamSol-PTM	Linear peptides with natural and non-natural residues	Sequence and monomer physicochemical descriptors; measured intrinsic solubility	Solubility prediction; retrospective with held-out evaluation	Explicit ncAA support within represented chemistry	M1/T1	Web server No public source-code repository identified	Endpoint is intrinsic solubility; transfer to formulation and unseen modifications requires new validation [73]
PeptideCLM	Cyclic peptides	Chemical language model on cyclic-peptide permeability data	Membrane diffusion prediction; task-specific retrospective split	Chemical representation supports cyclic chemistry	M1/T0	GitHub; Hugging Face	Scaffold similarity and applicability domain can dominate apparent performance [57,72]
PeptiVerse	Multi-class therapeutic peptides	Unified platform over multiple curated property datasets	Multi-property prediction; task-specific benchmark splits	Depends on endpoint and model representation	M1/T0	Hugging Face code/models Interactive Space	Broad coverage is valuable, but each endpoint retains its own data and domain limitations [53]
CAMP	Peptide–protein interactions	Peptide and protein sequence with multi-level attention; known interacting/non-interacting pairs	Interaction and binding-residue prediction; retrospective benchmark splits	No general explicit ncAA or cyclisation treatment	M1/T0	GitHub; Zenodo Downloadable local implementation	Negative-pair construction and protein-family leakage can inflate transfer claims [75]
ToxinPred2	Proteins and peptides (broad toxicity screen)	Sequence features and machine learning on curated toxin data	Toxicity classification; retrospective evaluation	Canonical alphabet	M0/T0	Web server; GitHub Standalone/data	Task is protein toxicity, not peptide-specific haemolysis, cytotoxicity or immunogenicity [82]
ProsperousPlus	Protease-susceptible peptides	Sequence scoring functions; protease-specific cleavage datasets; ensemble machine learning	Protease-specific cleavage prediction; retrospective benchmarking on independent test datasets	Canonical sequence features	M1/T0	GitHub/Public code, data and final models	Protease-specific cleavage prediction does not by itself establish stability in plasma, luminal or intracellular compartments [78]
NetMHCpan/NetMHCIIpan	Vaccine and epitope peptides	Pan-allele neural networks over binding-affinity and eluted-ligand data	Binding and presentation prediction; allele-stratified evaluation	Canonical alphabet	M1/T0	NetMHCpan server; NetMHCIIpan server Licenced download; no public source-code repository identified	Presentation is not immunogenicity; allele and pathogen sampling bias limits population claims [35]
***Prospective receptor-pharmacology models***							
Puszkarska et al.	GCGR/GLP-1R dual agonists	Sequence-function landscape from measured receptor activity libraries	Multi-receptor activity optimisation; experimental train/test design	Chemical format bounded by library design	M1/T1	GitHub Code/models/data	Campaign-specific transfer; receptor potency must still be connected to PK and efficacy [5]
Nielsen et al. (streaMLine)	GLP-1-related metabolic peptides	Sequence and high-throughput assay features from a large experimental library	Candidate prioritisation and optimisation; project/time-stage evaluation	Bounded by synthesised library chemistry	M1/T2	Proprietary platform No public source-code repository identified	The study reports receptor pharmacology, physicochemical profiling, pharmacokinetics, and efficacy in diet-induced obese mice, but not a class-relevant in vivo safety or tolerability assessment; T3 is therefore not assigned [6]

Note: Repository status was checked on 26 September 2026 against the cited article and author- or publisher-linked resources. “No public source-code repository identified” means that none was located through those sources by the review cut-off; it does not establish that no code exists. Web-server, model-hub, archived-record, or licenced-download access is reported separately and is not equated with publicly available source code.

## 6. Generative Design Across Therapeutic Peptide Classes

Generative models do not simply score a fixed library; they define a probability distribution from which new candidates can be sampled. This changes the evaluation problem. A predictor can be tested on held-out examples, whereas a generator must be assessed for chemical validity, uniqueness, diversity, novelty, control of the requested property, synthetic feasibility, and prospective experimental success. These criteria can conflict: a generator that produces highly diverse sequences may leave the domain of reliable activity prediction, while a conservative generator may reproduce close analogues of the training set.

Circular evaluation is the central risk. If the same activity predictor is used to guide generation and then to declare the generated peptides successful, the system can exploit errors in that predictor without improving real biology. Independent models, similarity analysis, chemical rules, structure-based checks, and uncertainty penalties can reduce this risk, but only prospective experiments can reveal whether the optimisation objective corresponded to the intended property. Table 5 lists representative generative and design methods with their evidence levels.

### 6.1. Sequence Generation and Foundation-Model Conditioning

The principal generative architectures search sequence space in different ways. Autoregressive language models generate one residue at a time, conditioning each choice on the partial sequence and any supplied property or target information. Masked-language models fill missing residues or entire peptide spans, which is useful for analogue optimisation and target-conditioned reconstruction. Variational autoencoders compress sequences into a continuous latent space in which nearby points tend to decode to related peptides. Diffusion models begin from a corrupted or random representation and learn to recover increasingly structured candidates through iterative denoising. These mechanisms are technically different, but all depend on the distribution and labels present in training data.

AMPs provide a representative case of conditional generation, but the organism and assay context, as well as the need for matched host-toxicity testing, are discussed in Section 2.4. Generative methods differ in how they condition and sample sequences, deepAMP couples template-conditioned sequence optimisation with an activity predictor, whereas diffusion and other language-model approaches use different sampling mechanisms [20,25,26,27], and should be compared by prospective novelty, hit rate, selectivity, and developability under the same experimental budget.

Target-conditioned generation changes the condition from a phenotype to a protein. PepMLM masks the whole binder region appended to a target sequence, whereas EvoBind2 uses iterative structure prediction to search for sequences and geometries compatible with the target [7,8]. Generative deep-learning pipelines can also combine sequence proposals with docking and molecular dynamics [9]. These methods are valuable because they direct the search toward one target rather than toward generic peptide-like statistics.

The target condition does not eliminate the need for negative information. A model trained mainly on positive peptide-target pairs may learn target-compatible motifs without learning selectivity against homologous proteins. In addition, a peptide generated for a target sequence may bind a region that is inaccessible in the native complex, membrane, or cellular state. Negative targets, competing interfaces, alternative conformations, and cellular context should therefore be included in model design or downstream filtering whenever the intended claim is target specificity.

### 6.2. Multi-Objective Optimisation and Pareto Reporting

Therapeutic peptide design is intrinsically multi-objective. Increasing hydrophobicity may improve membrane interaction or binding while reducing solubility and increasing nonspecific toxicity. Conformational restriction may improve affinity and protease stability but impair synthesis or trap an unfavourable conformer. Half-life extension can improve exposure while changing receptor access or tissue distribution. A single composite reward hides these trade-offs behind weights that are often chosen heuristically.

Pareto optimisation provides a more transparent view. A candidate is Pareto-optimal when no objective can be improved without worsening at least one other objective. Reporting the non-dominated set allows readers to see whether the model discovered a genuinely favourable region or merely shifted along an expected trade-off. Final candidate selection can then incorporate project priorities, but the weights and thresholds should be fixed before outcomes are known. Reporting only the winning sequence conceals both the trade-off and the degree of expert intervention.

Reinforcement learning (RL) is often used to optimise a generative policy against several predicted rewards. The oncology screen discussed above used RL to balance exploration of a large helical-peptide space with exploitation of promising regions [13]. RL can search efficiently, but it is especially vulnerable to reward hacking: the policy may move toward sequences for which a predictor is confidently wrong. Uncertainty-aware rewards, penalties for leaving the training domain, diversity constraints, and periodic experimental retraining are safeguards rather than optional refinements.

A convincing multi-objective study should therefore release the uncollapsed objective values for every prospectively tested candidate, report how the outcome changes when reward weights are varied, and state whether the selected candidates remained within the applicability domains of the component models.

### 6.3. Cyclic, Stapled, and Chemically Constrained Generation

Cyclic and stapled peptides require a generator to design more than residue order. It must specify monomer identity, stereochemistry, the positions and chemistry of the crosslink, and a topology that can be synthesised and can access a useful conformational ensemble. A sequence that would be favourable as a linear peptide may become strained or geometrically incompatible after closure. Conversely, the same residue sequence can yield distinct molecules when cyclized through different atoms.

Chemical-language models provide one route to this problem. HELM-GPT generates macrocyclic peptides in a notation that explicitly represents monomers and connections [71]. This helps maintain topology during generation and permits non-canonical building blocks within the represented vocabulary. However, syntactic validity does not establish that a macrocycle will form efficiently, adopt the intended conformation, or bind the target. Validity, cyclisation outcome, conformational behaviour, and biological function are separate endpoints.

Structure-aware systems address the geometric layer. RFpeptides designs protein-binding macrocycles by conditioning generation on the target, and experimental studies have confirmed high-affinity binders for selected cases [10]. This is compelling evidence for target recognition, but the accessible monomers and ring chemistries remain bounded by the design framework, and broader medicinal properties such as permeability, metabolic stability, and exposure must be optimised separately.

CycloPepper addresses a complementary bottleneck: whether a proposed therapeutic peptide can be cyclized successfully and under which conditions [79]. Its automated experimental dataset and prospective synthesis validation illustrate that makeability is itself a learnable design objective, which is the generative counterpart of the manufacturability argument in Section 5.3. Stapled peptides pose a similar requirement. Studies of cellular uptake show that increasing helicity alone is not sufficient; staple placement, hydrophobicity, and charge jointly influence internalisation and membrane-disruptive toxicity [4]. Generative systems should therefore connect structural constraint to experimentally measured pharmacology rather than optimise a secondary-structure proxy in isolation.

### 6.4. Generative Design for CPPs, Oncology, Vaccines, and Self-Assembly

The class-specific constraints established in Section 2.3, Section 2.5, Section 2.6 and Section 2.7 determine both the conditioning variables and the validation endpoints for generation. CPP models should account for cargo, cell type, intracellular destination, and productive delivery (Section 2.5), whereas oncology models should incorporate normal-cell selectivity and, for peptide–drug conjugates, the complete peptide–linker–payload construct (Section 2.3) [13,33]. Vaccine design is primarily a constrained selection-and-assembly problem and must extend beyond predicted MHC binding to presentation, reactivity, and population coverage (Section 2.6). Self-assembly models should condition on the experimental environment and intended morphology or material property rather than a generic binary aggregation label (Section 2.7) [38,39,40].

### 6.5. How to Interpret a Generative Peptide Study

De novo expression design covers several distinct achievements. A model may optimise close analogues of a known peptide, recombine motifs represented in its training set, or explore sequence and chemical space with no obvious local template. These modes are all useful, but they support different claims. Analogue design tests whether the model can navigate a defined medicinal-chemistry neighbourhood; broader generation tests whether it can leave that neighbourhood without losing activity or feasibility. deepAMP illustrates iterative template-based optimisation rather than template-free generation [20]. A manuscript should state which regime was used and quantify how far selected candidates lie from the examples that shaped the model.

The full candidate funnel is equally important. Generation may begin with thousands or millions of virtual sequences, but many will be duplicates, chemically invalid, outside the model’s applicability domain, difficult to synthesise, or unacceptable on safety and developability grounds. The informative denominators are therefore the numbers generated, rendered valid and unique, retained after prespecified filters, selected for synthesis, successfully produced and quality controlled, experimentally active, and ultimately qualified against all project criteria. Reporting only the final leads hides whether the algorithm enriched the experimental set or whether success depended mainly on extensive filtering and expert rescue. Failed synthesis, insolubility, aggregation, and assay interference define the practical design space and should remain visible.

Novelty must also be defined at the level relevant to the peptide class. Low global sequence identity may coexist with a conserved short pharmacophore, the same macrocyclic scaffold, or a nearly identical distribution of charge and hydrophobicity. Conversely, a new crosslink, stereochemical pattern, linker, or payload can create substantial chemical novelty without changing the displayed residue string. Sequence identity, motif similarity, chemical scaffold, topology, and nearest training neighbour should therefore be considered together. High novelty is not automatically desirable: as a candidate moves farther from represented chemistry, both prediction error and synthetic risk generally increase. A persuasive study shows how novelty, confidence, and experimental success trade off rather than presenting novelty as an isolated virtue.

Finally, predicted activity is not independent confirmation when the same model, or one trained on overlapping data, generated the candidate. The clearest measure of generative performance is experimental enrichment: how many candidates satisfy a predefined set of biological and developability requirements per unit of experimental effort, and how that yield compares with analogue search, nearest-neighbour ranking, diversity selection, or expert design under the same synthesis budget. This measure accommodates both conservative analogue optimisation and ambitious exploration while preventing the size of a virtual library, or the score of an internal oracle, from being mistaken for discovery.

## 7. Structure-Aware Peptide Design

Structure-aware methods are attractive because peptide activity often depends on three-dimensional recognition. AlphaFold2, RoseTTAFold, ESMFold, and AlphaFold3 have transformed protein and complex prediction, while ProteinMPNN, RFdiffusion, Chroma, and folding diffusion show how geometry can guide molecular design [63,83,84,85,88,89,90,91]. RoseTTAFold All-Atom extends structure prediction and design to assemblies containing proteins, nucleic acids, small molecules, metals, and covalent modifications [92]. Independent protein–peptide benchmarking further shows that performance depends strongly on input delimitation and evaluation against non-redundant complexes [93]. These advances provide powerful priors for peptide work, yet their success on globular proteins or well-represented complexes cannot be transferred automatically to short, flexible, chemically modified peptides.

Peptide-focused full-atom generators extend this lineage in different ways. PepFlow uses multimodal flow matching to co-design residue identity, backbone frames, and side-chain geometry, whereas PepGLAD uses geometric latent diffusion conditioned on a target binding site [86,87]. Both were evaluated primarily on computational benchmarks. By contrast, RFpeptides adapts the RFdiffusion-ProteinMPNN lineage to protein-binding macrocycles and includes prospective affinity and structural validation, although not pharmacokinetic or in vivo therapeutic evidence [10].

Rather than repeating the class-specific endpoints described in Section 2, it is helpful here to separate three structural questions that are often merged. First, can the peptide adopt a physically plausible pose on the target? Second, does that pose correspond to favourable and selective binding under the relevant conditions? Third, does binding produce the intended biological effect at achievable exposure? Structure prediction primarily addresses the first question. Docking, molecular dynamics, and newer affinity-predicting models can refine hypotheses about the first and second. None of these methods alone establishes the third.

### 7.1. Why Peptide-Protein Complexes Remain Difficult

Unbound peptides frequently populate broad conformational ensembles and may become ordered only after target engagement. Binding can proceed through conformational selection, induced fit, or a mixture of both. Multiple orientations may satisfy local contacts, and the target itself can reorganise. A prediction method that returns one complex necessarily compresses this landscape into a single hypothesis [94].

Peptides also provide weaker evolutionary signals than full-length proteins. Short linear motifs can recur by chance, and many therapeutic sequences have no natural homologues. Complex predictors may rely more heavily on learned structural patterns or training-set similarity than on co-evolution, which makes it important to examine whether a target or interface resembles examples seen during model development.

Biological context can change the accessible binding site. Membranes, cofactors, oligomeric states, post-translational modifications, partner proteins, and local pH may stabilise conformations absent from the input model. A peptide designed against an isolated soluble domain may encounter a different surface in the native receptor or multiprotein complex, and for intracellular targets, crowding and competition can further reduce effective binding.

Dedicated benchmarks such as PepPCBench and TopoDockQ improve method comparison by focusing on peptide-protein complexes [95,96]. They can reveal advances in pose quality or ranking, but a benchmark structure is usually a known binder under crystallographic or cryogenic conditions. Prospective discovery remains harder because the true negative space is poorly defined and affinity, selectivity, and cellular function must still be measured.

### 7.2. Non-Canonical Chemistry and Model Mismatch

Protein-centric structure predictors were trained predominantly on natural L-amino-acid polymers. AlphaFold3 and RoseTTAFold All-Atom can represent standard Chemical Component Dictionary entries for modified residues and ligands. Input support, however, should not be confused with validated accuracy for staples, D-rich macrocycles, backbone N-methylation, or non-peptidic linkers [83,92]. Confidence metrics were calibrated mainly on structures dominated by canonical chemistry, and there is little evidence that they retain the same meaning for unusual peptide chemotypes. A high interface-confidence score in this setting should therefore be treated as an untested extrapolation rather than as a reliable indicator, and coverage should be checked component by component rather than assumed from the model’s general accuracy.

Where a modification is genuinely unsupported, replacing it with a canonical surrogate can still be useful for generating an initial binding hypothesis, but the approximation must be explicit. A D-residue can invert side-chain orientation, N-methylation removes a hydrogen-bond donor, and a staple imposes long-range constraints. The surrogate model may therefore predict the wrong free-state ensemble, ring closure, or interface contacts, and the actual synthesised molecule should be modelled or experimentally challenged before a mechanistic conclusion is drawn.

Force-field coverage creates a related limitation for molecular dynamics. Parameters for non-canonical monomers and linkers may be unavailable or derived with inconsistent levels of theory, and simulation stability can then reflect the parameterisation rather than the molecule. Reports should specify residue templates, charge derivation, solvent and membrane models, restraints, and replicate trajectories, and should treat MD as a test of a defined model rather than as proof that the biological complex is correct.

### 7.3. A Defensible Structural Workflow

A defensible workflow begins by generating more than one hypothesis. Alternative peptide conformations, target states, binding sites, and orientations should be considered, particularly when no experimental motif is known. Predictions can be filtered using known mutational data, conserved interface residues, or competition with a natural partner, but these constraints should be kept separate from information used in final validation.

Local refinement and molecular dynamics can test whether key contacts persist and whether the proposed complex is compatible with the chosen environment. Results should be compared across starting poses and replicates rather than reported from one favourable trajectory. A stable simulation is not evidence of high affinity: force fields can stabilise incorrect contacts, and dissociation often occurs on timescales inaccessible to routine simulations.

Experimental design should follow directly from the structural hypothesis. Interface-disrupting peptide mutations, target mutations, scrambled or composition-matched controls, competition assays, and orthogonal binding methods can distinguish a specific pose from nonspecific association. If the peptide is intended to act in cells, target engagement and pathway rescue should be measured in addition to biochemical binding.

Model-confidence scores should be described as internal indicators rather than experimental observables: per-residue and interface scores estimate consistency within the model, not affinity, residence time, or selectivity. The appropriate conclusion from a predicted peptide-target complex is that a testable structural hypothesis has been generated.

## 8. Integrating Models with Experiments

AI becomes therapeutically meaningful when it changes which experiments are performed and increases the value obtained from a limited experimental budget. A closed design-make-test-learn cycle begins with a defined design space and target product profile, applies a model and a prespecified selection rule, synthesises and measures the selected candidates, and uses all outcomes to update the next cycle. Each step must be auditable, because hidden manual selection can contribute more than the formal algorithm.

Closed-loop design is not simply repeated prediction. The measurements obtained in one cycle should reduce uncertainty, expand the model’s domain, or improve the yield of qualified candidates in the next. As detailed for each peptide class in Section 2, a loop cannot learn solubility, toxicity, stability, or route-specific exposure if those properties are not measured; the experimental panel must include the liabilities most likely to prevent translation [18,97].

### 8.1. Prospective Candidate Selection and Matched Comparators

Prospective evaluation should lock the candidate pool, model version, objective functions, feasibility filters, and number of experiments before outcomes are available. These details define the denominator needed to calculate a hit rate, and reporting the best peptide without them prevents readers from assessing enrichment. Complete reporting matters most when synthesis fails: a chemically invalid or poorly soluble design is not missing data but evidence that the generative and filtering system did not capture an essential constraint.

The selected set should be compared with a meaningful baseline under the same budget. Random selection tests whether any intelligent ranking is useful; diversity selection tests whether broad coverage alone explains success; nearest-neighbour or simple physicochemical models test whether the complex model adds value; and expert selection measures performance against current practice. All arms must obey the same synthesis and feasibility constraints, otherwise the comparison favours the method that receives more human curation.

The primary outcome, hit rate above a prespecified threshold, best qualified candidate, Pareto hypervolume, reduction in uncertainty, or cost per candidate meeting a product profile, should match the programme and be declared before testing, so that a study cannot select whichever metric looks most favourable afterwards.

### 8.2. Active Learning and Experimental Information Gain

Active learning selects the next experiment by combining predicted value with uncertainty or diversity. Exploitation focuses on candidates expected to perform well, whereas exploration samples regions that are uncertain but potentially informative. A successful campaign usually needs both: pure exploitation can remain trapped near known scaffolds, while pure exploration consumes experiments without advancing the lead series.

Different acquisition functions encode different priorities. Uncertainty sampling targets cases on which the model disagrees; expected improvement favours candidates likely to exceed the current best; upper-confidence-bound methods combine predicted performance and uncertainty; diversity or batch methods avoid selecting near-duplicates. The acquisition function should be chosen in relation to the scientific goal rather than treated as an interchangeable algorithmic detail.

Performance should be evaluated across cycles using measures such as learning-curve area, calibration improvement, expansion of the applicability domain, reduction in prediction-interval width, number of qualified candidates per synthesis, and information gained per experiment. The self-assembling peptide and hydrogel studies discussed in Section 2.7 provide clear examples because iterative experimental measurements can be linked directly to changes in model performance and discovery yield [38,39,40]. The most credible demonstration is prospective and counterfactual: under the same experimental budget, the active-learning policy should outperform a non-adaptive or simpler selection strategy. Finding a good peptide after several cycles does not by itself show that adaptivity was responsible, and the acquisition history, including rejected and failed candidates, is needed to reconstruct that comparison.

### 8.3. Autonomous Agents, Reinforcement Learning, and Human Oversight

LLM-based agents can coordinate literature retrieval, database queries, property predictors, generative models, structural-analysis tools, and experimental planning. Their potential advantage is orchestration: a controller can invoke specialised tools, inspect outputs, and decide which operation to perform next. A peer-reviewed multi-agent protein-design system has demonstrated this modular strategy across retrieval, structure analysis, simulation, and result interpretation [98]. However, such evidence does not yet establish autonomous wet-laboratory peptide discovery, for which complete trajectories, human interventions, prospective synthesis, and experimental outcomes remain essential.

An agent inherits the limitations of every tool it invokes. A fluent explanation cannot rescue an uncalibrated toxicity model or a generator operating outside its chemical domain. Tool versions, prompts or policies, input data, model outputs, and manual overrides should be logged so that the trajectory can be reproduced, and the controller should be prevented from using final test data or changing objectives after outcomes are known.

Human oversight remains essential for defining the target product profile, recognising assay artefacts, evaluating synthetic feasibility, and setting safety boundaries; the relevant question is not whether humans are present but whether their contribution is visible. Claims of autonomy should therefore be graduated. A system may automate candidate generation, automate tool execution, or control the next prospective experiment within predefined limits. Full closed-loop autonomy would require the system to make and record those decisions, receive experimental results without selective filtering, and outperform matched baselines across locked cycles. Until then, agentic peptide design is best viewed as decision support and workflow engineering rather than as an independent scientific investigator.

## 9. From Computational Score to Therapeutic Evidence

Peptide–AI studies often place evidence of very different strength in the same narrative. A retrospective area under the curve, a favourable docking pose, synthesis of one active peptide, and efficacy in an animal model do not support the same claim. The difficulty in comparing such studies is not only that they stop at different points, but that they are strong along different axes. A paper may report a rigorously leakage-controlled, externally validated model and never synthesise a molecule. Another may report a mouse efficacy study built on a model evaluated with a random split. Both are legitimate contributions, both are incomplete, and neither is more advanced than the other in any single ordering.

The Peptide–AI Evidence Grid is not intended as a new universal reporting guideline or technology-readiness scale. Its methodological axis operationalises established validation and reporting principles [59,99], while its staged-evidence structure has precedents in Machine Learning Technology Readiness Levels (MLTRL) [100]. MLTRL follows an ML system from research through productisation and deployment; in contrast, the T axis used here grades evidence for the peptide molecule rather than deployment of the software. The narrower contribution is to keep computational rigour (M) and experimental/translational depth (T) orthogonal and claim-specific, and to define both axes for peptide chemistry, assay context, prospective selection, developability, and exposure. Progress on either axis cannot compensate for weakness on the other.

The Evidence Grid separates the two dimensions because in vivo evidence does not supersede methodological rigour. A claim can reach the highest translational level while the computational evaluation remains weak; in that case, the biology may be real while attribution to AI remains unsupported. The operational Grid therefore separates two axes. The methodological axis (M0–M3) describes how rigorously the computational component was evaluated. The translational axis (T0–T3) describes how far the resulting molecules were taken experimentally. Each defined claim-evidence unit receives an M/T pair. In Table 4 and Table 5, the pair assigned to each scored row refers to the principal claim represented in that entry; distinct claims within the same publication may warrant different pairs. Table 6 defines the levels.

### 9.1. Reading the Grid

The two axes generate sixteen combinations, and the interesting information is usually in the mismatch between them. Table 7 gives the archetype associated with each region and the interpretation it warrants.

Three readings deserve emphasis. First, the M0/T2-T3 corner is a recurrent source of overstatement in the literature, because a persuasive in vivo result licences a computational claim that the evaluation cannot support. Second, an M2/T0 assignment is not a weakness but a legitimate and necessary form of contribution; predictive-methods papers should be judged on the methodological axis and should not be criticised for lacking animal data they never claimed. Third, among the 25 peptide-specific entries assigned a discrete M/T pair in Table 4 and Table 5, none reached M3 (0/25; 0/11 in Table 4 and 0/14 in Table 5). The grouped Batra/Xu/Njirjak overview and the two general protein-model rows in Table 5 were not scored. Within this representative set, locked, comparator-controlled prospective selection therefore remains a major evidentiary gap and one of the most informative experiments the field could perform.

The M/T grid grades the evidence reported in the cited study; it is not a regulatory-development scale. Across the 25 scored entries, the translational distribution is 12 T0, eight T1, four T2, and one T3; the methodological distribution is four M0 and 21 M1 assignments, with no M2 or M3 assignment. The sole T3 entry remains a preclinical animal study [17]. To distinguish publication-level evidence from subsequent development status, Table 8 summarises three representative, publicly traceable clinical-stage candidates from Gubra-associated programmes. Candidate-level use of streaMLine is explicitly documented for ABBV-295 and HMB-003; for BI 3034701, the public sources support only a collaboration-level linkage to the platform and peptide-chemistry workflow.

Assignments are made per claim rather than per paper. A study may therefore receive M2/T3 for evidence concerning its lead molecule and M1/T1 for a broader platform-generalisation claim. The assignments in Table 4 and Table 5 refer to the principal claim represented by each scored row and are the author’s reading of the published record, offered as a worked demonstration rather than adjudicated scores. Grouped rows are not assigned a single score unless a uniform claim-level pair is supportable across their constituent sources. Several assignments remain approximate where a publication does not report split construction or candidate denominators in sufficient detail, which itself supports the reporting standards in Section 10.

### 9.2. Minimum Versus Ideal Evidence

Minimum evidence is the lowest level compatible with a particular statement. A computational generator can claim to have produced testable candidates when molecular validity, novelty, and domain have been assessed. It cannot claim therapeutic discovery without synthesis. A prospectively active peptide can support enrichment for an assay endpoint, but mechanism requires orthogonal target evidence, and therapeutic potential requires relevant developability and exposure.

Ideal evidence strengthens generality and attribution: blinded testing reduces experimenter bias, publication of all selected candidates permits hit-rate and calibration analysis, matched comparators establish whether AI added value, and replication across targets or laboratories tests transfer. Keeping minimum and ideal practice visibly distinct allows early methods to be reported fairly without overstating maturity. Neither axis is a development sequence that every study must complete. A self-assembling materials paper may legitimately stop at T2, since physical and functional characterisation is the relevant endpoint; a systemic receptor agonist generally requires T3 before an in vivo proof-of-concept claim is justified. The central principle is that each claim should be linked to the experiment most capable of falsifying it, and that the wording of the abstract should match the pair the study actually reached.

## 10. Reporting Standards for Peptide AI

Transparent reporting is necessary because peptide–AI performance depends on details that are easy to omit: molecular identity, assay conditions, data relatedness, candidate filters, uncertainty, and manual intervention.

### 10.1. Relation to Existing Reporting Frameworks

Neither the Evidence Grid nor the peptide-specific checklist is intended to replace existing reporting or readiness frameworks. The current DOME framework and registry organise reporting for supervised machine learning in biology around Data, Optimisation, Model, and Evaluation [59]; complementary reproducibility standards specify the artefacts required for a life-science machine-learning result to be repeatable [60]; TRIPOD + AI provides a 27-item checklist for reporting studies that develop, validate, or update diagnostic, prognostic, monitoring, or screening prediction models [99]; and the FAIR principles govern how the underlying data and models should be deposited so that they remain findable and reusable [105]. These frameworks should be applied where they fall within a study’s scope; the peptide-specific checklist below complements rather than replaces them. Direct operational resources are available through the DOME checklist and guidelines, the DOME Registry, the TRIPOD + AI checklist, and the FAIR Guiding Principles.

What those frameworks do not cover in peptide-specific detail is the chemistry-and-assay layer that determines whether a peptide result is interpretable. Three requirements are specific to this field. First, molecular identity is not established by a sequence string: stereochemistry, terminal chemistry, crosslink position and topology, salt form, and conjugation must be recorded, because two records with the same displayed sequence can be different molecules and two records with different sequences can be near-duplicates. Second, relatedness must be computed on chemistry as well as sequence, since scaffold, ring system, linker, and payload create leakage that percentage identity does not detect. Third, the label is a peptide-context pair, not a peptide property: organism, cell line, cargo, barrier, allele, and assay protocol are part of what was measured and must travel with the value. Table 9 converts these recurring issues into a checklist, distinguishing the minimum information needed to interpret a result from stronger practices that support reproducibility and transfer.

Table 9 is designed to function as a stand-alone checklist that can be completed during model development and manuscript preparation. Its purpose is to make peptide-specific decisions visible within the article and associated deposited datasets, without requiring readers to reconstruct them from dispersed narrative descriptions.

### 10.2. Benchmark Standards and Thresholds

Beyond the split conventions set out in Section 3.1, a basic benchmark needs baselines that span increasing complexity. A simple composition or physicochemical model reveals whether a deep network is merely exploiting charge or hydrophobicity differences; nearest-neighbour and conventional machine-learning baselines test the value of representation learning; and for generative studies, memorisation and novelty analyses should be accompanied by an independent evaluator and, ultimately, prospective assays.

Universal potency, toxicity, or selectivity thresholds would be misleading because peptide classes and assays differ. Standardisation should focus on process: a versioned dataset, explicit chemistry, cleaning and deduplication rules, fixed splits, baseline algorithms, appropriate metrics, calibration tests, and an applicability-domain statement. The dynamic range and precision of the assay should be reported so that model error can be compared with experimental variability. Metric choice should follow the endpoint: imbalanced classifiers need precision-recall analysis in addition to receiver-operating curves; continuous endpoints require both rank metrics, which assess prioritisation, and absolute-error metrics, which assess quantitative prediction; confidence intervals should reflect biological replicates and repeated model training; and thresholds for binary decisions must be chosen without consulting the final test set.

Benchmarks should be versioned, because repeated tuning to an unchanged public test set gradually converts it into training information. Hidden, periodically refreshed, or time-split test sets reduce this effect, and code, preprocessing, split membership, and model versions should be archived so that later improvements can be distinguished from changes in the data pipeline. Dedicated resources are emerging: PepPCBench provides a framework for peptide-protein complex prediction, PeptiVerse supports multiple peptide-property tasks, applicability-domain studies in cyclic-peptide permeability show how evaluation can be tied to chemical coverage, and PepBenchmark may further support comparable preprocessing across canonical and non-canonical tasks [53,54,57,95]. None removes the need to inspect endpoint definitions and relatedness for the particular claim.

A major gap in current benchmark suites is prospective evaluation. A mature benchmark ecosystem would allow models to be submitted against locked candidate pools, with synthesis and assays performed by an independent laboratory and complete outcomes released after evaluation. Such designs are more expensive than retrospective leaderboards, but they provide a direct route to populating the M3 column of Table 7 and thereby to test the decision problem that therapeutic peptide AI is intended to solve.

## 11. Outlook: Testable Priorities for the Next Generation of Peptide AI

1. **Complete, versioned data.** Datasets should record monomer structures, stereochemistry, termini, crosslinks, conjugation, assay conditions, and failed measurements in machine-readable form. Precompetitive sharing or federated learning may be needed to make consistent industrial measurements usable without disclosing proprietary records [50].
2. **Test representations and transfer.** Sequence-only, chemical-graph, and ensemble-aware models should be compared on matched datasets containing controlled changes in D-residues, N-methylation, staples, cyclisation, or linkers. Multimodal and foundation models require ablation, missing-modality tests, target-family holdouts, scaffold shifts, and calibrated abstention to establish transfer to new chemistry or biology.
3. **Make generation and explanation auditable.** Generative studies should predefine rewards and domain penalties, report full Pareto fronts and every prospectively tested candidate, and evaluate enrichment against matched baselines. Explanations should predict the effect of controlled perturbations rather than rely only on visually plausible attention maps.
4. **Treat manufacturability and regulation as design objectives.** Coupling difficulty, epimerisation risk, cyclisation yield, purification burden, and sequence-related impurities should be modelled alongside activity. Current regulatory guidance provides explicit requirements for impurity thresholds, higher-order structure, biological activity, and innate immune-response testing [81].
5. **Benchmark closed-loop and agentic systems.** Active-learning systems should be evaluated by information gained, qualified candidates produced, or time and cost saved relative to locked baselines, with tool provenance and human overrides disclosed. Autonomy should be claimed only when prospective decisions are made within declared boundaries and the system’s contribution can be separated from expert intervention [98].
6. **Connect structural hypotheses to mechanism and translation.** Structure-aware design will continue to improve, particularly for macrocycles and target-conditioned binders, while all-atom methods increasingly accommodate modified residues and other chemical components [83,92]. Predicted structures should be tested using alternative conformations, negative controls, affinity measurements, cellular target engagement, and exposure-response. Peptide-programme digital twins remain a longer-term objective requiring linked measurements from the complete chemical construct through formulation, exposure, pharmacodynamics, and disease response, with calibrated uncertainty.

## 12. Conclusions

AI for therapeutic peptide discovery is not a single discipline but a family of design problems linked by the interaction of sequence, chemistry, conformation, biological context, and translation. A balanced account of this breadth cannot be achieved by treating every peptide as the same type of sequence. Receptor agonists, PPI inhibitors, membrane-active peptides, delivery systems, antigens, conjugates, macrocycles, and self-assembling materials require different inputs, endpoints, and falsifying experiments.

The methods reviewed span protein-scale language and generative systems (ProtGPT2, ProGen/ProGen2, and ESM-2/ESM3), structure-conditioned design or prediction (ProteinMPNN, RFdiffusion/RFpeptides, and AlphaFold 3), and peptide-focused full-atom generators (PepFlow and PepGLAD). Across all three groups, evidence must be judged at the peptide-specific endpoint rather than inferred from protein-level performance.

The central technical lesson is that representation and evaluation are inseparable. Protein language models are powerful for canonical sequence patterns; chemical graphs and hierarchical notations are needed when topology and modifications define the molecule; and ensemble or multimodal approaches are needed when conformation and context dominate. No representation, however sophisticated, converts a predicted score or a plausible structure into experimental evidence.

The central translational lesson is that models should be judged by the decisions they improve, and that this judgement requires two questions to be kept apart: how well was the model evaluated, and how far were its molecules taken. The Peptide–AI Evidence Grid used here is a peptide-specific synthesis of established reporting and maturity concepts, and its utility lies in making that separation explicit. Applying it to the representative studies reviewed here indicates that the 25 scored entries comprise twelve T0, eight T1, four T2, and one T3 assignment, with no M3 assignment in Table 4 and Table 5. Similarity- and scaffold-aware benchmarks, calibrated uncertainty, complete prospective reporting, orthogonal mechanism, class-specific developability and manufacturability, pharmacokinetic and in vivo evidence, and equal-budget comparators define the path forward. The grid and the accompanying checklist are intended to make that path explicit, auditable, and progressively more difficult to overstate.

## Figures and Tables

**Table 1:** Therapeutic peptide classes, AI tasks, and decisive experimental evidence.

Peptide Class	Design Objective	Representative AI Tasks	Decisive Experimental Evidence
Endocrine and metabolic receptor agonists	Potency and signalling bias at a defined receptor; prolonged exposure; dosing convenience	Sequence-activity modelling, library prioritisation, multi-receptor optimisation, PK-aware design	Receptor pharmacology, selectivity panels, stability, PK/PD and efficacy in disease-relevant models
Target-specific PPI and intracellular inhibitors	Disrupt or stabilise a defined interface, often with intracellular delivery	Target-conditioned generation, motif completion, inverse folding, docking and affinity/selectivity prediction	Orthogonal binding, mutagenesis, cellular target engagement, permeability and pathway rescue
Oncology and tumour-targeting peptides	Tumour-selective binding or killing; delivery of imaging agents or payloads	Target prediction, anticancer prioritisation, receptor-ligand design, conjugate optimisation	Tumour-versus-normal selectivity, target engagement, biodistribution, payload release, efficacy and tolerability
Phenotype-driven bioactive peptides	Desired phenotype without a single established molecular target, including antimicrobial or antiviral activity	Activity classification/regression, de novo generation, proteome mining, species-aware ranking	Matched activity and host-toxicity assays, resistant strains, mechanism studies, PK and in vivo infection models
Cell-penetrating and delivery peptides	Cell or tissue entry, oral or transmucosal absorption, and delivery of a defined cargo	CPP classification/generation, cargo- and cell-context modelling, endosomal-escape and permeability optimisation	Cargo-dependent uptake, intracellular bioavailability, epithelial transport, systemic exposure, localisation, toxicity and in vivo distribution
Vaccine, epitope, and immunomodulatory peptides	Elicit or redirect a defined immune response against a selected antigen	Binding and presentation prediction, immunogenicity ranking, neoantigen prioritisation, multi-epitope construct design	Mass-spectrometry-confirmed presentation, T-cell reactivity in patient-matched cells, in vivo immunisation and response durability
Self-assembling and material-forming peptides	Programmed assembly into fibres, hydrogels or functional nanostructures	Assembly prediction, sequence generation, morphology/property optimisation	Biophysical assembly, rheology or morphology, function, biocompatibility and stability
Cyclic, stapled and chemically modified peptides	Conformational preorganization, protease resistance, permeability or improved target engagement	Graph/atom-level learning, ensemble modelling, topology-aware generation and synthesis prediction	Chemical identity, topology, conformational ensemble, permeability, stability, binding and synthesis yield

**Table 2:** Representative approved peptide therapeutics: amino acid sequences and chemically defining features.

Peptide and Therapeutic Class/Use	Sequence (N → C)	Defining Chemistry and Connectivity	Source
Semaglutide Acylated GLP-1 receptor agonist; type 2 diabetes	H[Aib]EGTFTSDV SSYLEGQAAK* EFIAWLVRGR G	Aib at chain position 2 (GLP-1 position 8); Arg at chain position 28 (GLP-1 position 34); K* is chain Lys20 (GLP-1 Lys26), whose ε-amino group is acylated through ADO–ADO–γ-Glu to 1,18-octadecanedioic acid; free C-terminal carboxylate	[41]
Bivalirudin Direct thrombin inhibitor; anticoagulation during percutaneous coronary intervention	[dF]PRPGGGGNG DFEEIPEEYL	D-Phe1; otherwise unmodified linear peptide with free termini (the formulated salt is not part of the sequence)	[42]
Enfuvirtide HIV-1 gp41 fusion inhibitor; treatment-experienced HIV-1 infection	Ac-YTSLIHSLIE ESQNQQEKNE QELLELDKWA SLWNWF-NH_2_	All residues are L-amino acids; N-terminal acetylation and C-terminal carboxamide	[43]
Ziconotide CaV2.2 (N-type calcium-channel) blocker; severe chronic pain by intrathecal administration	CKGKGAKCSR LMYDCCTGSC RSGKC-NH_2_	C-terminal amide; three disulfides: Cys1–Cys16, Cys8–Cys20, and Cys15–Cys25	[44]
Daptomycin Cyclic lipopeptide antibacterial; serious Gram-positive infections	Dec-W[dN]DTG[Orn]D[dA]DG[dS][(2S,3R)-3-MeGlu][Kyn]	N-decanoyl group; D-Asn2, D-Ala8, D-Ser11; L-Orn6, (2S,3R)-3-methyl-L-Glu12, L-kynurenine13; 10-residue macrolactone closed between the Thr4 side-chain oxygen and the Kyn13 C-terminal carbonyl	[45,46]
Lanreotide Somatostatin-receptor agonist; acromegaly and gastroenteropancreatic neuroendocrine tumours; self-assembling prolonged-release depot	[d-2-Nal]CY[dW]KVCT-NH_2_	D-2-naphthylalanine1, D-Trp4, C-terminal amide, and Cys2–Cys7 disulfide	[47,48]
Lutetium (^177^Lu) dotatate Somatostatin-receptor-targeted radioligand; SSTR-positive gastroenteropancreatic neuroendocrine tumours	[^177^Lu-DOTA]-[dF]CY[dW]KTCT-OH	DOTA attached by an amide bond to N-terminal D-Phe and chelating ^177^Lu; D-Trp4; Cys2–Cys7 disulfide; free C-terminal carboxylate	[49]

Note: Sequences are written from N to C. Unmarked residues are L-amino acids. Bracketed d-residues have D stereochemistry, and bracketed names denote non-canonical residues or conjugated groups. Ac, acetyl; Aib, α-aminoisobutyric acid; ADO, 8-amino-3,6-dioxaoctanoic acid; Dec, decanoyl; Orn, ornithine; 3-MeGlu, 3-methylglutamic acid; Kyn, kynurenine; DOTA, 1,4,7,10-tetraazacyclododecane-1,4,7,10-tetraacetic acid. NH_2_ denotes a C-terminal carboxamide; OH denotes a free C-terminal carboxyl group. Asterisks identify side-chain derivatisation. Salt forms and excipients are omitted.

**Table 3:** Representative data resources and their appropriate use in therapeutic peptide AI.

Resource, Version and URL	Coverage	Key Annotations or Representation	Principal Value	Important Caveat
***General therapeutic-peptide resources***				
THPdb2 Version: v2 URL: https://webs.iiitd.edu.in/raghava/thpdb2/	Approved therapeutic peptides and proteins	Indication, target, sequence and therapeutic annotation	Anchors models and reviews in clinically used modalities	Approved set is small and selected; not a negative or discovery benchmark [51]
Peptipedia v2.0 Version: v2.0 URL: https://app.peptipedia.cl/	Broad peptide sequence and function space	Integrated sequence and functional annotations	Cross-class discovery and database navigation	Heterogeneous sources and labels require task-specific curation [52]
PeptiVerse Version not stated URL: https://huggingface.co/spaces/ChatterjeeLab/PeptiVerse	Multi-property therapeutic peptide prediction	Curated tasks and unified predictive platform	Supports cross-property baselines and standardised access	Task coverage and split suitability must be checked per endpoint [53]
***Benchmark and class-specific resources***				
PepBenchmark Version not stated URL: https://github.com/ZGCI-AI4S-Pep/PepBenchmark	Canonical and non-canonical peptide ML tasks	Standardised datasets, preprocessing and model-family baselines	Emerging cross-task comparison and reproducible pipelines	ICLR 2026 conference paper; benchmark performance is not prospective therapeutic validation, and each task still requires domain-specific scrutiny [54]
CancerPPD2 Version: v2.0 URL: https://webs.iiitd.edu.in/raghava/cancerppd2/	Anticancer peptides and proteins	Activity, source and cancer-related annotations	Oncology-focused hypothesis generation	Mechanistic and assay heterogeneity; anticancer label may not imply selectivity [11]
PDCdb Version not stated URL: https://pdcdb.idrblab.net/	Peptide–drug conjugates across disease areas	Peptide, linker, payload, activity and pharmaceutical annotations	Composite-conjugate representation and translation-oriented data retrieval	Literature-derived endpoints are heterogeneous; related constructs and source overlap require leakage control [16]
CPPsite 2.0 Version: v2.0 URL: https://webs.iiitd.edu.in/raghava/cppsite/	Experimentally validated cell-penetrating peptides	Sequence, cargo and experimental context where available	Delivery-focused training and benchmarking	Uptake assays and cargo contexts vary; cytosolic delivery may be unresolved [32]
IEDB Version: v3.10.0 URL: https://www.iedb.org/	Immune epitopes across pathogens, allergens and cancer	Binding, elution, T-cell and B-cell assay records with allele and host context	Standard substrate for epitope and immunogenicity models	Strong allele and pathogen sampling bias; assay type must be modelled, not pooled [36]
CyclicPepedia Version not stated URL: https://www.biosino.org/iMAC/cyclicpepedia/	Natural and synthetic cyclic peptides	Topology, sequence, structure and function annotations	Knowledge base for cyclic peptide chemistry	Coverage and notation consistency vary across sources [55]
CycPeptMPDB Version: v1.1 URL: http://cycpeptmpdb.com/	Cyclic peptide membrane permeability	Structures and permeability measurements	Task-specific permeability modelling	Assay conditions and chemical-series similarity constrain generalisation [56,57]
CREMP Version: v1.0.1 URL: https://doi.org/10.5281/zenodo.8010582	Macrocycle conformer-rotamer ensembles	3D ensembles for defined macrocyclic chemotypes	Ensemble-aware geometric learning	Computed ensembles are not equivalent to experimental populations [58]
DRAMP 4.0 Version: v4.0 URL: https://dramp.cpu-bioinfor.org/	AMPs with translational annotations	Activity, target organism, toxicity and development-related metadata	Large curated AMP resource with explicit translational orientation	Protocol heterogeneity and overlapping sources still require leakage control [21]
DBAASP v3 Version: v3.0 URL: https://dbaasp.org/	Antimicrobial and cytotoxic peptides	Activity, structure and toxicity annotations	Matched AMP activity and host-liability analyses	Missing values and assay heterogeneity limit direct pooling [22]
CAMPR4 Version: v4 URL: https://camp.bicnirrh.res.in/	Natural and synthetic AMPs	Sequences, structures and curated metadata	Independent source for cross-database evaluation	Overlap with other AMP databases must be removed before external testing [23]

Note: Version identifiers are those declared by the resource or its associated release record. “Version not stated” indicates that no formal public version identifier was exposed at the time of access. All URLs were accessed on 25 September 2026.

**Table 5:** Representative generative, structure-based, and closed-loop design methods, with evidence level as defined in Section 9.

Method	Peptide Class	Representation and Training Data	Task and Evaluation	ncAA/Topology Handling	Evidence (M/T)	Code/Repository	Principal Limitation
***Foundation and target-specific design models***							
ProtGPT2; ESM-2/ESM3; ProGen/ProGen2	General protein foundation models	Masked, autoregressive, or multimodal models trained mainly on large natural-protein corpora	Protein representation and conditional or unconditional generation; protein-level retrospective and selected functional tests	Canonical protein alphabet; no explicit therapeutic-peptide topology	n.a./n.a.	ProtGPT2; ESM-2 ESM3-open; ProGen/2	Protein-level performance does not establish validity for short, modified therapeutic peptides [63,64,67,68,69]
ProteinMPNN; RFdiffusion; AlphaFold 3	General structure-aware protein models	Protein backbones, inverse folding, diffusion, and all-atom biomolecular-complex representations	Sequence design, backbone generation, and complex prediction; general protein or biomolecular benchmarks	Mostly canonical for design; AlphaFold 3 can represent standard CCD components	n.a./n.a.	ProteinMPNN; RFdiffusion AlphaFold 3 (weights under separate terms)	Foundational for peptide workflows, but not direct evidence of peptide affinity or therapeutic performance [83,84,85]
PepMLM	Target-conditioned binders	Masked protein language model conditioned on target sequence	Binding-peptide generation; retrospective plus prospective synthesis and functional testing	Primarily canonical sequence generation	M1/T2	GitHub; Hugging Face	Predicted structures are supporting hypotheses; intracellular access and off-target binding require independent evidence [7]
EvoBind2	Target-specific binders	Evolutionary and structure-aware iterative design over protein structural information	Peptide binder design; benchmark plus prospective SPR validation	Linear and cyclic; topology support is method-dependent	M1/T1	GitHub; Zenodo	Performance depends on target structure and conformational assumptions; only a subset of designs bound [8]
RFpeptides	Protein-binding macrocycles	Structure-conditioned geometric deep learning on protein structures	De novo macrocycle binder design; design benchmarks plus prospective in vitro binding	Explicit macrocyclic topology; chemistry-bounded	M1/T1	RFdiffusion GitHub Archived release	High-affinity binding does not establish permeability, PK or in vivo efficacy [10]
PepFlow (Li et al.)	Target-specific peptide binders	Multimodal flow matching for receptor-conditioned full-atom sequence-structure co-design	Sequence-structure co-design, fixed-backbone sequence design, and side-chain packing; test clusters held out after 40% peptide-sequence clustering	Full-atom canonical residues; modified chemistry and cyclic topology not established	M1/T0	GitHub Code/data/pretrained weights	Computational validation only; prospective synthesis, binding, and selectivity remain untested [86]
PepGLAD	Target-specific peptide binders	Geometric latent diffusion for binding-site-conditioned full-atom sequence-structure co-design	Sequence-structure co-design and binding-conformation generation; cross-target test with receptors separated at 40% sequence identity	Full-atom canonical residues; modified chemistry and cyclic topology not established	M1/T0	GitHub Code/data/trained weights	Computed structural and binding proxies require prospective experimental validation [87]
Chen et al.	Target-specific peptide inhibitors	Generative learning plus structure modelling and molecular dynamics	Generate and structurally refine inhibitors; pipeline-specific evaluation with prospective support	Mostly sequence-level chemistry; target-format dependent	M1/T2	Code Ocean; Zenodo	MD and docking refine hypotheses but do not establish affinity or mechanism alone [9]
deepAMP	AMPs	Pretrained peptide language model with task-specific AMP-activity and penetration-optimisation modules	Template-conditioned optimisation in two iterative design rounds; 29 candidates synthesised; antibacterial, haemolysis, resistance, and mouse wound-infection testing	Mostly canonical linear peptides	M1/T2	GitHub/Code, data, trained models	Selection and host-toxicity controls determine therapeutic relevance [20]
***Class-specific generation and experimental workflows***							
Latent-diffusion AMP model	AMPs	Latent diffusion conditioned on activity over AMP sequence/activity data	Diverse AMP generation; retrospective plus prospective in vitro testing	Mostly canonical linear peptides	M1/T1	GitHub; Zenodo	Candidates are optimised against learned activity proxies; independent evaluation needed [25]
LLAMP	Species-aware AMPs	Language model with explicit organism context; species-specific measurements	Activity prediction and optimisation; retrospective species-aware evaluation plus reported tests	Primarily canonical sequences	M1/T1	GitHub; Model	Protocol and strain metadata remain critical; not a structural-refinement method [19]
Pandey et al.	Anticancer peptides	Activity-cliff-informed oracle and scalable reinforcement learning over a large helical-segment library	Prioritise bioactive peptides; campaign-specific screen plus prospective cancer-versus-normal testing	Primarily canonical amidated peptides	M1/T1	GitHub; Zenodo	Training centred on a related chemical series; mechanism, PK and in vivo efficacy not established [13]
HELM-GPT	Macrocyclic peptides	Generative pre-trained transformer over HELM notation	De novo macrocycle generation; retrospective validity, novelty and property metrics	Explicit monomer and connection notation	M0/T0	GitHub Code/data	Syntactic validity is not cyclisation outcome, conformational behaviour or binding [71]
CycloPepper	Therapeutic cyclopeptide synthesis	Chemistry-aware ML on cyclisation reactions and outcomes	Predict cyclisation and optimise synthesis; retrospective plus prospective experimental validation	Explicit cyclic chemistry within training scope	M1/T1	GitHub; Zenodo Web platform	Synthesis success is distinct from biological activity; transfer to unseen reactions must be calibrated [79]
LightCPPgen	CPPs	Explainable ML and genetic algorithm over CPP sequence/activity datasets	Rational CPP generation; retrospective evaluation only	Primarily canonical; bounded chemistry	M0/T0	GitHub Code/data/notebooks/model	Feature explanations require perturbational validation and cargo-matched testing [33]
Hsueh et al.	Multifunctional delivery peptide and PDC	ML-guided sequence engineering over peptide feature data	Optimise melanin binding, cell penetration and sustained ocular delivery; prospective in vitro and rabbit pharmacology	Canonical linear peptide conjugated to brimonidine	M1/T3	Code/data archive Project page	Strong project-specific demonstration; transfer to other cargos, tissues and routes requires new calibration [17]
Batra/Xu/Njirjak frameworks	Self-assembling peptides and hydrogels	Sequence models, active learning and hybrid experimental descriptors	Assembly prediction and design; study-specific retrospective and prospective loops	Mostly short canonical peptides	n.a./n.a.	Batra archive; Njirjak GitHub Xu: no study-specific public code/model repository identified	Macroscopic properties depend on solvent, processing and timescale; single-structure scores are insufficient [38,39,40]

Note: General protein models are included as methodological context and are not assigned peptide-specific M/T levels; peptide-focused methods are graded only on the evidence reported in the cited primary study. The Batra/Xu/Njirjak row is retained as a grouped descriptive overview but is not assigned a single M/T pair and is excluded from all scoring denominators because its constituent studies do not support one uniform claim-level assignment. Repository status was checked on 26 September 2026 against the cited articleand author- or publisher-linked resources. Web-server, model-hub, archived-record, or licensed-download access is reported separately and is not equated with publicly available source code. n.a. indicates not available.

**Table 6:** The Peptide–AI Evidence Grid: definitions of the methodological and translational axes.

Level	Minimum Evidence	Ideal Strengthening	Claim Supported	Overclaim to Avoid
**Methodological axis, rigour of computational evaluation**				
M0, unvalidated or self-referential	Random split only, no leakage control, no baseline, or evaluation against the same predictor used for generation	Any leakage-controlled held-out evaluation	A computational hypothesis exists	Reporting cross-validation accuracy as evidence of generalisation
M1, similarity-aware retrospective	Held-out evaluation with sequence and scaffold leakage control, relevant baselines, and uncertainty on the reported metric	Multiple similarity thresholds, chemistry- and target-family splits, released code and split membership	The model generalises within a defined retrospective domain	Describing retrospective enrichment as prospective discovery
M2, external validation and calibration	Frozen model tested on a deduplicated external source, later time period or distinct laboratory, with calibration and a declared applicability domain	Pre-registered analysis plan and calibrated abstention	Transfer beyond the development dataset is demonstrated	Calling a second overlapping database “independent”
M3, locked prospective protocol	Candidate pool, model version, objectives, filters and sample size fixed before outcomes; matched random, diversity or expert comparator under equal budget	Multi-site replication, cost and time analysis, complete released trajectory	The AI selection procedure itself improves decisions	Attributing success to the algorithm when experts silently re-ranked candidates
**Translational axis, depth of experimental evidence**				
T0, in silico evidence only	Retrospective model evaluation or in silico candidate assessment, with the evaluated domain and molecular validity stated where applicable	Applicability-domain analysis and an independent predictor or physics-based sanity check where candidates are proposed	A computational relationship or testable candidate hypothesis has been established within a defined in silico domain	Presenting retrospective performance or untested candidates as experimental discovery
T1, prospective synthesis and primary assay	Predeclared candidates synthesised, identity and purity confirmed, primary endpoint measured with controls, all selected candidates reported	Replicate dose–response and blinded assay execution	The method prospectively yields molecules with measurable activity	Reporting only the successful candidates or selecting post hoc
T2, orthogonal functional evidence	Prospective experimental evidence beyond the primary assay, using class-appropriate orthogonal measurements of mechanism, selectivity, developability, or material function	Mutational or rescue experiments, counter-target panels, formulation-relevant measurements, or orthogonal structural and materials characterisation, as applicable	The claimed activity or material function is supported by class-appropriate orthogonal evidence	Equating a single phenotype, predicted pose, or assembly readout with mechanism or translational readiness
T3, exposure, efficacy and tolerability in vivo	Exposure, dosing, pharmacodynamics, efficacy and safety interpreted together in a relevant model	Independent replication, exposure-response and tissue distribution	The candidate has in vivo proof of concept under stated conditions	Calling in vitro potency therapeutically validated

**Table 7:** Reading the evidence grid: study archetypes by methodological and translational level.

	M0	M1	M2	M3
**T3**	Biology may be real; the AI contribution is unsupported	Credible programme; computational credit is campaign-specific	Strong translational study with defensible modelling	No example identified in the representative set
**T2**	Orthogonal functional result with unsupported computational attribution	Common profile of the strongest current design papers	Well-founded design study	Rare; the appropriate target for the field
**T1**	Hit found; attribution to the model unclear	Standard prospective generative study	Prospective study with demonstrated transfer	Would establish a genuine selection advantage
**T0**	Computational hypothesis only; generalisation remains unsupported	Standard retrospective methods paper	Benchmark-grade predictive model	Not applicable without synthesis

**Table 8:** Representative, publicly traceable clinical-stage peptide candidates linked to or associated with AI-enabled discovery programmes as of the September 2026 cut-off.

Candidate/Programme	Documented AI/ML Involvement or Programme Linkage	Development Stage at Cut-Off	Publicly Reported Outcome/Status
ABBV-295 Long-acting amylin analogue	Discovered by Gubra using the streaMLine platform [101]	Phase 2; NCT07752979	Company-reported Phase 1 multiple-dose data showed dose-dependent weight reduction up to 9.8% at 12 weeks and favourable safety and tolerability; Phase 2 was initiated [101]
BI 3034701 GLP-1/GIP/NPY2 triple agonist	Developed in a Gubra–Boehringer Ingelheim collaboration that Gubra describes as combining its streaMLine platform and peptide chemistry with Boehringer Ingelheim’s clinical-development capabilities [102]	Phase 2; NCT07662122	Phase 2 was initiated for dose-finding and broader efficacy and safety evaluation; no numerical clinical outcomes were reported in the cited public sources [102,103]
HMB-003 Peptide plasmin inhibitor	Discovered in the Gubra-Hemab collaboration using streaMLine [104]	Phase 1; NCT07798505	The first healthy volunteer was dosed on 17 September 2026; the trial was ongoing, and no clinical outcomes were available at the cut-off [104]

Note: This is a representative, not exhaustive, clinical development-status table. The cited company sources explicitly document candidate-level streaMLine involvement for ABBV-295 and HMB-003. For BI 3034701, they describe a Gubra-Boehringer Ingelheim collaboration combining streaMLine and peptide chemistry but do not document platform use for that individual programme.

**Table 9:** Peptide-specific reporting checklist for AI-assisted therapeutic peptide studies, to complement applicable DOME, TRIPOD + AI, and FAIR guidance.

Domain	Minimum Requirement	Ideal Practice	Applies to	Report or Release
***Study definition, data, and chemistry***				
Task and intended use	Define biological endpoint, unit, target/organism/cell context, route and the decision supported	Prospective product profile and decision threshold	Both	Protocol, label definition and intended-use statement
Data provenance and eligibility	List sources, inclusion/exclusion, collection dates and assay harmonisation	Persistent identifiers and machine-readable provenance	Both	Dataset card and exact version
Chemistry and notation	Retain stereochemistry, termini, modifications, connectivity and conjugation	HELM, graph or atom-level representation plus validation rules	Both	Canonicalised structures and rejected records
***Validation and model reliability***				
Duplicates and relatedness	Remove exact duplicates and disclose conflicting measurements	Cluster by sequence plus scaffold, topology and target family	Both	Cluster assignments and nearest neighbours
Splitting	Use a split appropriate to the claim; state the similarity criterion, threshold and rationale; disclose inter-partition similarity	Declared primary threshold with two sensitivity analyses, plus scaffold and time splits where applicable	Predictive; generator evaluators	Frozen train/validation/test membership
Baselines and statistics	Compare with simple and relevant baselines; report uncertainty and replicate structure	Nested tuning, confidence intervals and prospective comparator arms	Both	Code, seeds and statistical plan
Calibration and applicability domain	Report calibrated confidence or interval coverage and a domain rule	Conformal or ensemble uncertainty, abstention curves and local error maps	Both	Reliability plots and out-of-domain flags
***Generation, experimental validation, and translation***				
Generation validity and novelty	Report generated, valid, unique, filtered and selected counts, with nearest training neighbours	Scaffold novelty, memorisation tests and an independent evaluator	Generative	All candidate identifiers and filters
Multi-objective optimisation	Predefine objectives, constraints and selection rule; report trade-offs	Full Pareto front or hypervolume and robustness to reward weighting	Generative/optimisation	Uncollapsed objective values for every tested candidate
Structural assessment	Present confidence as uncertainty; include alternative poses and negative controls; state whether the modelled chemistry is within the predictor’s validated scope	Ensemble and refinement sensitivity, mutational constraints, blinded benchmark	Structure-aware	Inputs, model versions and complete structural hypotheses
Prospective synthesis and assays	Confirm identity and purity; dose–response; biological replicates; positive and negative controls	Blinded testing and independent laboratory replication	Both	All tested sequences including failures
Mechanism and selectivity	Use orthogonal target-engagement or mechanistic assays and relevant counter-screens	Competition, mutagenesis, rescue and cellular engagement	Both	Raw and processed data with predefined criteria
Developability and manufacturability	Measure class- and route-relevant solubility, stability, toxicity and synthetic feasibility	Formulation, permeability, serum binding, impurity profile and cost-of-goods assessment	Both	Conditions, units and acceptance thresholds
PK, PD and in vivo	Connect exposure, dose, pharmacodynamics, efficacy and tolerability	Exposure-response, tissue distribution and independent replication	Translational claims	Full dosing and formulation detail with prespecified endpoints
Closed-loop performance	Report each cycle, acquisition rule, manual overrides and an equal-budget baseline	Information gained per experiment, cost and time, multi-site replication	Active learning and agents	Complete trajectory, failures and model versions
Evidence level	State the M/T pair reached for each principal claim	Per-claim assignment with justification	Both	Statement in abstract and discussion

## Data Availability

No new data were created or analysed in this study. Data sharing is not applicable to this article.
